# Modulating biodiesel yield and purification with plant-derived hydrophobic iron oxide nanocatalysts

**DOI:** 10.1039/d5na00879d

**Published:** 2026-01-08

**Authors:** Kaouthar Ahmouda

**Affiliations:** a Department of Process Engineering and Petrochemistry, Faculty of Technology, University of El Oued El Oued 39000 Algeria ahmouda-kaouthar@univ-eloued.dz; b Renewable Energy Research Unit in Arid Zones, University of El Oued El Oued 39000 Algeria

## Abstract

This study investigates the impact of the hydrophobicity of iron oxide (FeNP) nanocatalysts on biodiesel production and post-reaction purification. FeNPs were green synthesized using distinct hydrophobic extracts of *Rosmarinus officinalis* (ROS), *Matricaria pubescens* (MAT), *Juniperus phoenicea* (JUN), and *Artemisia herba-alba* (ARM), whose phytochemical contents showed large variations in hydrophobic polyphenols (flavonoids (TCF): 209.50–353.75 mg AGE per g; condensed tannins (TCCT): 853.04–871.45 mg CE per g). Biodiesel production was performed under optimized conditions (ethanol-to-oil volume ratio, 3 : 1; catalyst loading, 0.20 wt%; 65 °C), and the biodiesel/purification performance was evaluated using FTIR and UV-vis analysis of retained (Gly_Bio_) and free glycerol (Gly_free_). The results show a strong positive correlation between extract hydrophobicity and catalytic efficiency. The most hydrophobic extract (ROS: TCF = 353.75 ± 1.02 mg AGE per g; TCCT = 871.45 ± 0.89 mg CE per g) produced FeNPs that achieved the highest biodiesel yield (92.60 ± 1.12%), glycerol separation efficiency (98.30 ± 0.01%), and ester content (98.25%), with minimal glycerol contamination (1.46 ± 0.21 mM; 152.70 µg g^−1^). Conversely, FeNPs synthesized from the least hydrophobic extract (ARM: TCF = 209.50 ± 0.89 mg AGE per g; TCCT = 853.04 ± 0.83 mg CE per g) exhibited significantly lower biodiesel yield (81.42 ± 2.03%), purification efficiency (88.60 ± 0.63%), and ester content (89.09%), with higher glycerol contamination (8.69 ± 0.32 mM; 909.40 µg g^−1^). ANOVA (*p* < 0.0001) and Tukey's HSD confirmed statistically significant differences between the four green nanocatalysts. Spectroscopic analysis further supported these findings, showing reduced OH bands from glycerol and enhanced 3,5-diacetyl-1,4-dihydrolutidine (DDL) signals in samples purified with more hydrophobic catalysts, demonstrating effective oxidation and removal of glycerol. Overall, nanocatalysts derived from hydrophobic extracts retained less glycerol and promoted cleaner phase separation, while less hydrophobic extracts favored stronger glycerol surface interactions, reducing biodiesel purity. This work highlights the novel link between extract hydrophobicity, nanoparticle surface chemistry, and biodiesel quality, providing a green strategy for designing plant-based nanocatalysts capable of producing EN 14214-compliant biodiesel (≤1.91 mM glycerol). The economic assessment underscores the commercial promise of this method. The production cost for biodiesel was calculated to be $1.12 per kg, a figure that is highly competitive and partly attributable to the use of a hydrophobic ROS-FeNP catalyst. This property significantly reduces downstream purification costs by facilitating the effortless separation of glycerol. Coupled with a low catalyst cost of $6.538 per kg and compliance with international EN 14214 standards, this methodology highlights significant potential for large-scale industrial implementation.

## Abstract 2

This study investigates how plant-extract-mediated hydrophobicity of iron oxide nanocatalysts affects biodiesel production efficiency and post-reaction purification.

## Graphical abstract

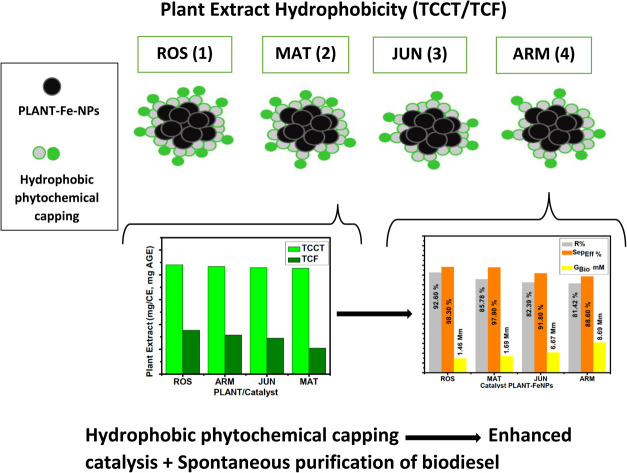

## 1 Introduction

Biodiesel is a renewable and biodegradable fuel derived from vegetable oils, animal fats, or waste cooking oils through a transesterification reaction. In this process, triglycerides (TGs) react with an alcohol such as ethanol,^1,2^ in the presence of a catalyst, to produce alkyl fatty acid esters (FAEEs, biodiesel) and glycerol as a byproduct.^3–6^ Biodiesel offers several environmental advantages, including reduced greenhouse gas emissions and improved biodegradability compared to conventional fossil fuels.^7–9^ In addition, its renewable nature helps to decrease dependence on petroleum resources. The transesterification process, influenced by several factors such as catalyst type, is crucial in determining the yield and quality of biodiesel.^3,10,11^

Glycerol is a major by-product formed during the transesterification of TGs in biodiesel production, which typically represents approximately 200 µg g^−1^ of the final product mixture.^12–14^ Its presence significantly affects the quality of biodiesel by increasing viscosity, reducing combustion efficiency, and potentially causing injector fouling in engines if not properly removed. In addition, residual glycerol can retain water and alcohol, leading to phase separation and microbial growth during storage.^15,16^ Therefore, efficient glycerol separation is essential to meet biodiesel quality standards such as EN 14214.^17^ Common methods for glycerol removal include gravity settling,^18^ where glycerol naturally separates due to its higher density, centrifugation to accelerate phase separation, and washing steps using water or mild acids to extract the remaining polar impurities.^19^ Advanced techniques such as membrane filtration^20,21^ or adsorption using solid resins can also enhance glycerol purification,^22^ especially in continuous processing systems. Ensuring maximum glycerol separation is crucial not only for biodiesel quality but also for downstream valorization of glycerol as a feedstock for other industrial applications.

These conventional methods for separating glycerol from biodiesel are often associated with several inconveniences and limitations that justify the search for catalysts that promote easier *in situ* separation. These drawbacks include: time-consuming separation, high water consumption and emulsification, energy-intensive and costly equipment, membrane fouling and lifespan issues, and loss of biodiesel during purification. These limitations drive research toward hydrophobic or phase-selective catalysts that enhance *in situ* phase separation. Such catalysts facilitate the spontaneous migration of free glycerol into a separate phase, enabling direct separation from the biodiesel layer and minimizing post-reaction purification steps, thus improving process efficiency and sustainability. We hypothesize that the hydrophobic phytochemicals in plant extracts will impart hydrophobicity to the synthesized FeNPs, which will enhance their catalytic performance by improving mass transfer and, critically, facilitate the separation of the glycerol by-product, reducing post-reaction purification burdens.

The use of green synthesized catalysts in biodiesel production has gained significant attention due to their environmental compatibility, cost-effectiveness, and alignment with the principles of circular bioeconomy.^23–27^ Conventional chemical catalysts often rely on hazardous reagents, generate toxic by-products, and require high temperatures or pressures, making them unsuitable for large-scale sustainable processes.^28^ In contrast, green synthesis employs plant extracts rich in phytochemicals that act simultaneously as reducing, stabilizing, and capping agents, eliminating the need for toxic chemicals and minimizing waste generation. These phytochemical mediated nanoparticles exhibit unique surface functionalities that enhance catalytic activity, selectivity, and post-reaction separation efficiency. Furthermore, the use of renewable biological resources ensures a lower environmental impact and reduces the operational cost of catalyst preparation. Therefore, plant-based nanocatalysts represent a promising alternative for the development of cleaner, safer, and more energy efficient biodiesel production technologies.^28,29^

Iron oxide nanoparticles (FeNPs) have gained significant attention as catalysts in biodiesel production due to their unique properties, including high surface area, strong catalytic activity, and eco-friendly synthesis potential.^30–33^ FeNPs can be synthesized using green methods using plant extracts, reducing dependence on toxic chemicals and aligning with sustainable practices.^34,35^ They exhibit robust stability in diverse reaction environments, making them viable catalysts for scalable biodiesel synthesis. Surface engineering through functionalization enables precise tuning of their physicochemical properties, allowing customized catalytic activity and improved interfacial interactions to optimize performance under specific operating conditions.^31,36,37^

The catalyst plays a crucial role in facilitating the transesterification process, primarily due to its physicochemical properties,^38^ particularly its hydrophobic nature. Hydrophobic catalysts improve reaction efficiency by improving the interaction between TGs and the alcohol phase while minimizing water interference, which can lead to soap formation and reduced biodiesel yield.^39–42^

Several studies in the literature have investigated the impact of the hydrophobic properties of the catalyst on biodiesel production. Karimi *et al.*^39^ explored the development and application of a nanosized hydrophobic sulfated mordenite catalyst for the production of biodiesel using an electrochemical approach. They reported that nanohydrophobic sulfated mordenite is an effective and stable catalyst for the electrochemical production of biodiesel from neem seed oil. Its hydrophobic properties and nanoscale size contribute to improved catalytic performance, making it a promising candidate for sustainable biodiesel production. Similarly, Sreeprasanth *et al.*^40^ investigated the synthesis and application of hydrophobic solid acid catalysts for the efficient conversion of biomass-derived feedstock to biofuel and lubricant. They demonstrated that surface hydrophobicity significantly enhances the performance of solid-acid catalysts in biomass conversion reactions. By repelling water and improving compatibility with hydrophobic reactants, these catalysts offer a robust and reusable platform for the sustainable production of biofuel-based lubricants. Furthermore, Zhang *et al.*^41^ investigated the design and application of novel catalysts for biodiesel synthesis. Specifically, the study focused on acidic polymeric ionic liquids (PILs) enhanced with hydrophobic regulators to improve the catalytic performance and stability in biodiesel production processes. The study demonstrated that tailoring the hydrophobic properties of acidic PILs can significantly improve their efficiency and stability as catalysts in biodiesel production. The enhanced hydrophobicity not only mitigates the adverse effects of water in the reaction medium but also contributes to the catalyst's reusability and overall process sustainability. Khandan *et al.*^42^ investigated the eco-friendly modification of fumed silica to develop a hydrophobic basic heterogeneous catalyst for biodiesel synthesis. This study demonstrated that green hydrophobization of fumed silica is a promising strategy to tailor effective basic heterogeneous catalysts for biodiesel production. By enhancing water resistance and compatibility with hydrophobic feedstock, the approach improves both catalytic efficiency and operational stability.

While seminal studies have conclusively demonstrated that engineered hydrophobicity enhances catalytic performance,^39–42^ they have primarily relied on post-synthetic chemical modifications to impart this property. For instance, Karimi *et al.*^39^ utilized sulfated mordenite, while Zhang *et al.*^41^ employed hydrophobic regulators in polymeric ionic liquids. This reliance on synthetic chemistry creates a significant research gap: the potential of using the intrinsic hydrophobic phytochemicals from a plant extract during a one-pot green synthesis to autonomously create a hydrophobic catalyst surface remains largely unexplored. Furthermore, the reported benefits of these hydrophobic catalysts are almost exclusively discussed in the context of improving reaction kinetics and biodiesel yield. Crucially, their role in passively modulating post-reaction phase behavior, specifically in facilitating the critical *in situ* separation of glycerol from biodiesel, has been systematically overlooked.

This study bridges this gap by demonstrating, for the first time, that the innate hydrophobic character of a plant extract can be directly imprinted onto FeNPs during phytosynthesis, dictating their performance in a dual capacity. We reveal a direct correlation between plant-derived hydrophobicity and enhanced glycerol separation efficiency, alongside high biodiesel yield. This approach moves beyond simply using plants as a generic green reducing agent; it strategically leverages their biochemical diversity as a design tool to create multifunctional nanocatalysts. We hypothesize that hydrophobic phytochemicals, such as flavonoids and condensed tannins, form a non-polar capping layer on the FeNPs, which not only improves mass transfer at the triglyceride–alcohol interface but also promotes phase separation by destabilizing the biodiesel–glycerol emulsion. Therefore, this work introduces a novel, sustainable strategy for integrated biodiesel production and purification, where the catalyst is intrinsically designed to drive the transesterification reaction while simultaneously easing the downstream purification burden, offering a pathway to more efficient and economically viable biofuel synthesis. We demonstrate this using four distinct FeNP green nanocatalysts synthesized from *Rosmarinus officinalis* (ROS-FeNPs), *Matricaria pubescens* (MAT-FeNPs), *Juniperus phoenicea* (JUN-FeNPs), and *Artemisia herba-alba* (ARM-FeNPs), which exhibit a gradient of hydrophobic properties. This approach provides a sustainable and integrated strategy for biodiesel production, where the catalyst actively facilitates the reaction and passively supports downstream purification, thereby reducing the need for energy-intensive separation steps. The selection of the synthesis route and feedstock was strategic, as justified in Table 1.

**Table 1:** Comparative justification of feedstock and catalyst synthesis routes

Aspect	Options	Rationale for selection
Catalyst synthesis route	Chemical synthesis	Uses toxic reagents; requires high energy; generates hazardous waste
	Microbial synthesis	Requires sterile conditions; time-consuming; may have low yield
	Plant-mediated green synthesis (this study)	Rapid, cost-effective, scalable; uses phytochemicals as non-toxic reducing and capping agents; allows plant biochemical signatures to be imprinted onto the nanoparticle surface^38^
Feedstock type	Non-edible or waste oils	Sustainable but highly variable in composition, complicating standardized catalyst evaluation
	Refined sunflower oil (this study)	Provides a consistent, reproducible, and impurity-free triglyceride source that allows accurate evaluation of catalyst hydrophobicity without interference from feedstock variability^43^

The transesterification reaction was conducted under standardized conditions using ethanol as the alcohol source. Biodiesel yield and glycerol separation efficiency were monitored to evaluate the performance of each FeNP catalyst. To investigate the glycerol separation efficiency of the catalysts, the post-reaction mixture was first allowed to settle for 24 hours, enabling natural phase separation by density. The free glycerol that settled at the bottom was then decanted from the biodiesel phase. The glycerol content in both this separated fraction and the portion retained within the biodiesel was quantified using a consistent oxidative colorimetric method. This involved the oxidation of glycerol to formaldehyde with sodium periodate, followed by colorimetric detection with Nash reagent, allowing for a direct comparison of glycerol removal efficacy across the different FeNP catalysts. The results revealed that FeNPs synthesized with more hydrophobic plant extracts facilitated a cleaner phase split, likely due to enhanced surface interactions and polarity contrast, enabling more efficient glycerol demixing. Furthermore, statistical analyses (ANOVA and Tukey's test) confirmed significant differences in biodiesel yield and glycerol separation efficiency across the different FeNP catalysts, directly correlating with the hydrophobicity of the mediating extract. This correlation was further supported by FTIR and UV-vis characterization, which indicated that the type of FeNPs influenced both the transesterification mechanism and the miscibility of products.

The remainder of this paper is organized as follows: the next section (Materials and methods) details the preparation and phytochemical characterization of the plant extracts, the green synthesis and characterization of the FeNPs, and the protocols for biodiesel production and analysis. Section 3 (Results and discussion) presents and discusses the findings, including the characterization of the plant extracts and FeNPs, the performance of the different catalysts in biodiesel yield and glycerol separation, the statistical analysis of the results, reusability study, and cost assessment of the nanocatalyst and biodiesel. Finally, Section 4 (Conclusion) summarizes the key findings and highlights the broader implications of this work for sustainable biodiesel production.

## 2 Materials and methods

This section describes the materials, equipment, and experimental methods used in the study, including the preparation and characterization of aqueous plant extracts, the ethanolic transesterification of triglycerides, and the evaluation of FeNP performance in biodiesel production and purification.

### 2.1 Materials

#### 2.1.1 Chemicals used for plant extract phytocomposition analysis

*Artemisia herba-alba*, *Rosmarinus officinalis*, *Matricaria Pubescens*, *Juniperus phoenicea*, aluminum trichloride (AlCl_3_, 99%, Sigma Aldrich), gallic acid (C_7_H_6_O_5_, 98%, Biochem Chemopharma), hydrochloric acid (HCl, 35%, Biochem Chemopharma), and catechin (C_15_H_14_O_6_, 99%, PubChem).

#### 2.1.2 Chemicals used in the ethanolic transesterification of TGs, characterization, and quantification of biodiesel and glycerol

Reagents for the transesterification reaction: vegetable oil (commercially available refined sunflower oil, used as the triglycerides source), ethanol (C_2_H_5_OH, 99.8%, Sigma-Aldrich), and the four plant-based FeNP nanocatalysts. Reagents used for detection of free and retained glycerol: sodium metaperiodate (NaIO_4_, 99%, Sigma-Aldrich); acetylacetone reagent, consisting of acetylacetone (2,4-pentanedione, C_5_H_8_O_2_, 99%, Sigma-Aldrich), glacial acetic acid (CH_3_COOH, 100%, Sigma-Aldrich), and ammonium acetate (CH_3_COONH_4_, 98%, Sigma-Aldrich); Nash reagent, consisting of acetylacetone, ammonium acetate, and sodium hydroxide (NaOH, pellets, 98%, Merck) for formaldehyde detection through the formation of 3,5-diacetyl-1,4-dihydrolutidine (DDL, yellow complex).

Reagents used for quantification of ester content in biodiesel: biodiesel samples, hydroxylamine hydrochloride (NH_2_OH, HCl, 99%, PubChem), ethyl oleate (CH_3_(CH_2_)7CH=CH(CH_2_)7COOC_2_H_5_, 98%, Sigma-Aldrich), methanol (CH_3_OH, >99.8%, Sigma-Aldrich), and *n*-hexane (C_2_H_5_OH, 99.8%, Sigma-Aldrich). Reagents used for determining the density of free glycerol: glycerol (C_6_H_14_, 99.5%, Sigma-Aldrich) and distilled water.

Reagents used for quantification of AV and FFA% in biodiesel: biodiesel samples, phenolphthalein indicator (C_20_H_14_O_4_, 99%, ROK Chem), ethanol (C_2_H_5_OH, 99.8%, Sigma-Aldrich), potassium hydroxide (KOH, (≥85%) pellets, Sigma-Aldrich), isopropanol (C_3_H_8_O, 99.7%, Merck), hydrochloric acid (HCl, 35%, Biochem Chemopharma), and toluene (C_7_H_8_, 99.5%, Sigma-Aldrich).

#### 2.1.3 Apparatus and methods used in the characterization of plant extract, biodiesel, and glycerol phases

A Fourier transform infrared spectrometer (FTIR, Nicolet iS5, Thermo Fisher Scientific) was utilized to analyze biodiesel, glycerol retained in biodiesel, and free glycerol separated from biodiesel. FTIR spectra were recorded between 500 and 4000 cm^−1^. UV-vis spectroscopy (Shimadzu UV-1800s) operating in the range of 200–900 nm was employed to detect the DDL (likely a specific analytical marker) indicator of retained and free glycerol, and quantify ester content.

### 2.2 Methods

This section outlines the experimental procedures for biodiesel production: protocols for biodiesel synthesis, including transesterification, and post-reaction steps. Biodiesel characterization: quality assessment through physicochemical analyses (*e.g.*, kinematic viscosity, density, and ester content) and spectroscopic methods. Glycerol separation and detection: techniques for isolating glycerol from the biodiesel phase and quantifying retained and free glycerol (*e.g.*, FTIR and UV-vis for DDL indicator analysis).

#### 2.2.1 Plant extract preparation and green synthesis of FeNPs

Aqueous extracts of *Artemisia herba-alba*, *Rosmarinus officinalis*, *Matricaria pubescens*, and *Juniperus phoenicea* were prepared for phytochemical characterization and FeNP synthesis. The fresh plant material was washed, air-dried, and ground to a fine powder. The powder was mixed with double-distilled water (1 : 10 w/v ratio) and agitated for 24 hours at room temperature. The slurry was filtered through muslin cloth, followed by 0.22 µm membrane filtration to obtain clear extracts, which were stored at 4 °C until use. FeNPs were synthesized using an eco-friendly, sustainable approach, where plant bioactive compounds (*e.g.*, polyphenols and flavonoids) acted as both reducing and capping agents. In a typical synthesis, 200 mL of plant extract was mixed with 100 mL of 0.4 M FeCl_3_ solution and stirred at 70 °C for 1 hour. The resulting precipitate was collected, washed, dried, and annealed at 500 °C for 2 hours to enhance crystallinity.^44^ The synthesized FeNPs were characterized using multiple techniques: XRD (phase identification, 2*θ* = 10–80°), FTIR-ATR (functional groups and crystallinity), SEM (surface morphology), and UV-vis spectroscopy (optical properties and band gap energy). All analyses were performed under ambient conditions.

#### 2.2.2 Plant extracts and phytochemical analysis

Aqueous extracts of *Rosmarinus officinalis*, *Artemisia herba-alba*, *Matricaria pubescens*, and *Juniperus phoenicea* were prepared as described in our previous work.^44^ The total antioxidant capacity (TAC) and polyphenol content of these extracts have been reported earlier and are briefly summarized here for comparison. In the present study, we extend the phytochemical characterization by additionally quantifying flavonoids and condensed tannins as hydrophobic compounds using standard colorimetric assays (AlCl_3_ and catechin-based methods, respectively). Full methodological details and calibration data for these new assays are provided in this work, while summarized results for all phytochemical parameters are presented in Table 2.

**Table 2:** Calculated total contents of polyphenols (TCP), flavonoids (TCF), condensed tannins (TCCT), and total antioxidant activity (TAC) of plant aqueous extracts

Extract	TCP (mg ACE)	TAC (mg per g DPE)	TCF (mg AGE)	TCCT (mg CE)
ROS	294.94 ± 0.93	358.34 ± 1.46	353.75 ± 1.02	871.45 ± 0.89
MAT	237.11 ± 1.04	181.45 ± 0.80	314.58 ± 1.04	867.33 ± 0.68
JUN	181.22 ± 1.06	228.15 ± 1.12	289.58 ± 0.93	858.11 ± 0.96
ARM	310.84 ± 1.43	249.56 ± 0.86	209.50 ± 0.89	853.04 ± 0.83

##### 2.2.2.1 Measurement of the total content of flavonoid

To determine the total flavonoid content (TFC) in aqueous extracts of *Artemisia herba-alba* (L.) and *Rosmarinus officinalis* (L.), 1 mL of a 2% aqueous aluminum chloride (AlCl_3_) solution was mixed with 1 mL of plant extract (0.2 mg mL^−1^). After 10 minutes of reaction, the absorbance was measured at a wavelength of 415 nm against a blank, following the method described in ref. 35. Gallic acid was used as the standard, and the results were expressed as milligrams of gallic acid equivalent per gram of dry plant extract (mg AGE/g extract). The flavonoid content was quantified using a linear calibration curve constructed with gallic acid solutions at concentrations ranging from 0.16 to 0.40 mg mL^−1^. The calibration equation obtained was *Y* = 7.3873*X* + 0.0271, with a correlation coefficient of *R*^2^ = 0.99.

##### 2.2.2.2 Measurement of the total content of condensed tannins

The condensed tannin content (TCCT) in aqueous extracts of *Artemisia herba-alba* (L.) and *Rosmarinus officinalis* (L.) was determined using the method described in ref. 35. A volume of 10 µL of plant extract solution (0.5 mg mL^−1^) was mixed with 120 µL of a 4% catechin aqueous solution. Subsequently, 60 µL of concentrated hydrochloric acid (HCl) was added to the mixture. The resulting red solution was allowed to stand for 15 minutes at room temperature, and the absorbance was measured at 500 nm. The condensed tannin content was expressed as milligrams of catechin equivalent per gram of dry plant extract (mg CE/g dry extract). Quantification was based on a calibration curve constructed using catechin solutions with concentrations ranging from 0.05 to 0.5 mg mL^−1^. The linear regression equation obtained was *Y* = 1.9033*X* − 0.0018, with a correlation coefficient of *R*^2^ = 0.9969.

#### 2.2.3 Structural, morphological, and optical properties of FeNPs

FeNPs were synthesized *via* an eco-friendly route using plant extracts as reducing and stabilizing agents, according to our established protocol.^44^ Extracts were mixed with FeCl_3_ solution and heated under stirring, followed by washing, drying, and annealing at 500 °C for 2 h. Detailed reaction conditions and mechanism are available in ref. 44.

Structural, morphological, and optical properties of FeNPs were analyzed using the same techniques reported previously.^44^ Briefly, crystalline phases were identified by XRD (Rigaku Miniflex 600, Cu Kα, 2*θ* = 10–80°), morphology by SEM (FEI Quanta 250), functional groups by FTIR (Shimadzu IR-Infinity, 500–4000 cm^−1^), and optical band gap by UV-vis spectroscopy (Shimadzu 1800, 200–900 nm). Crystallite size and lattice parameters were estimated using the Scherrer equation^45^ and standard models, while direct/indirect band gaps were determined by Tauc plots. Detailed procedures are provided in ref. 44; only essential findings are included here (Tables 3, 4 and Fig. 2–6) adapted from our previously published work.^44^

**Table 3:** Crystallographic data obtained from XRD patterns of four Fe-NPs annealed at 500 °C for 2 h. For α-Fe_2_O_3_ orthorhombic crystal cells (JCPDF file 01-089-0599), the standard *d*_104_ = 2.68695 Å at around 2*θ* = 33.19°, and standard lattice parameters are *a* = 5.0320 Å, *b* = 5.0320 Å, and *c* = 13.7330 Å. For γ-Fe_2_O_3_ cubic crystal cells (JCPDF file 00-039-1346), the standard *d*_311_ = 2.51770 Å at around 2*θ* = 35.63°, and standard lattice parameters are *a* = *b* = *c* = 8.3515 Å (ref. 44)

Sample	Phase	Quantity (%)	Diameter (nm)	Lattice parameters
ROS-Fe	γ-Fe_2_O_3_	75	23.5694	*a* = *b* = *c* = 8.3534 Å
ROS-Fe	α-Fe_2_O_3_	25	29.3379	*a* = 4.36566 Å, *b* = 4.36566 Å, *c* = 13.77657 Å
ARM-Fe	γ-Fe_2_O_3_	70	23.5682	*a* = *b* = *c* = 8.3607 Å
ARM-Fe	α-Fe_2_O_3_	30	29.3368	*a* = 4.36272 Å, *b* = 4.36566 Å, *c* = 13.78393 Å
JUN-Fe	γ-Fe_2_O_3_	66	23.5719	*a* = *b* = *c* = 8.3482 Å
JUN-Fe	α-Fe_2_O_3_	34	29.3413	*a* = 4.35438 Å, *b* = 4.36566 Å, *c* = 13.74545 Å
MAT-Fe	γ-Fe_2_O_3_	62	23.5724	*a* = *b* = *c* = 8.3464 Å
MAT-Fe	α-Fe_2_O_3_	38	25.8310	*a* = 4.36017 Å, *b* = 4.36566 Å, *c* = 13.77880 Å

**Table 4:** Optical band gap energies of α/γ-Fe_2_O_3_ NP samples annealed at 500 °C for 2 h (ref. 44)

Sample	Average *D* (nm)	*E*_g,dir_ (eV)	*E*_g,ind_ (eV)
ARM-α/γ-Fe_2_O_3_	29.3368/23.5682	2.91	1.82
ROS-α/γ-Fe_2_O_3_	29.3380/23.5695	2.88	1.80
MAT-α/γ-Fe_2_O_3_	29.0831/23.5719	2.77	1.71
JUN-α/γ-Fe_2_O_3_	29.3413/23.5724	2.66	1.61

Calculations of crystallite size, lattice constants, and band gap energy followed standard models (Scherrer equation, orthorhombic/cubic lattice formulas, and Tauc relations), as outlined in ref. 44. For completeness, the equations are not repeated here; readers are referred to the earlier work for methodological details.

#### 2.2.4 Green biodiesel production *via* transesterification using various phyto-nanocatalysts: characterization of biodiesel and glycerol phases

This section outlines the experimental procedures used for the phytosynthesis of biodiesel from TGs in vegetable oil through ethanol-based transesterification, catalyzed by various phyto-derived FeNPs. The methodology includes detailed descriptions of the materials, equipment, and reaction conditions. The transesterification process, facilitated by phytosynthesized catalysts, is followed by purification and analytical evaluation of the resulting biodiesel and glycerol phases.

##### 2.2.4.1 Green production of biodiesel *via* transesterification using various phyto-catalysts

The synthesis of biodiesel using phyto-derived FeNPs was performed through a standardized transesterification protocol. Initially, 0.1 g (0.20 wt%) of the iron oxide nanocatalyst was dispersed ultrasonically in ethanol under continuous stirring. This ethanol–catalyst dispersion was subsequently mixed with sunflower oil at an ethanol-to-oil volume ratio of 3 : 1. The resulting mixture was then subjected to ultrasonic treatment at 65 °C for 30 minutes to enhance catalytic efficiency and promote homogeneous mixing. Upon completion of the reaction, the mixture was transferred to a rotary evaporator to remove excess ethanol, thereby reducing the moisture content.

The mixture was centrifuged to separate and recover the solid catalyst (FeNPs), then washed with warm water and transferred to a separating funnel and left undisturbed for 24 hours to allow separation of the biodiesel (organic phase) from the free glycerol phase (aqueous phase).

##### 2.2.4.2 Catalyst recovery and regeneration

After the transesterification reaction, the solid iron oxide nanocatalyst was separated from the biodiesel–glycerol mixture *via* centrifugation at 5000 rpm for 10 minutes. The recovered catalyst was then purified to remove adsorbed organic species and regenerate its active surface. This regeneration protocol involved washing the catalyst sequentially with ethanol and hexane, followed by drying in an oven at 80 °C for 2 hours. The purified, dried ROS-FeNP catalyst was then reused in the subsequent five transesterification cycles under identical reaction conditions to evaluate its reusability and stability. The ROS-FeNP catalyst demonstrated significant catalytic potency up to the fifth run with >90% yield.

##### 2.2.4.3 Physicochemical characterization of biodiesel and its retained and free glycerol

The biodiesel layer was collected and analyzed to confirm its formation and purity. The biodiesel produced was characterized by measuring retained glycerol (Gly_Bio_), density (*d*_bio_), kinematic viscosity (*ν*_bio_), and ester content, which are critical indicators of fuel quality. Ensuring that the values fall within the international standard limits (*e.g.*, EN 14214) confirms the suitability of biodiesel for use as an alternative fuel and supports its compliance with established fuel standards.

##### 2.2.4.4 Evaluation of ester content within biodiesel samples

The ester content in biodiesel samples was determined *via* a hydroxamic acid derivatization method.^46^ The protocol begins with the preparation of the reagents. First, the hydroxylamine reagent is prepared by dissolving 5 g of hydroxylamine hydrochloride in 20 mL of distilled water, followed by the addition of 15 mL of 12% (w/v) sodium hydroxide, and dilution to 100 mL with water. Separately, the ferric chloride reagent is prepared by dissolving 1 g of FeCl_3_ in 100 mL of 0.1 M hydrochloric acid. For the standard curve, a stock solution of ethyl oleate (1 mg mL^−1^) is prepared by dissolving 0.1 g of the ester in 100 mL of *n*-hexane. Serial dilutions of this stock are made to generate standards ranging from 0.1 to 1.0 mg mL^−1^, with a solvent-only blank used for baseline correction.

In the reaction procedure, 1 mL of each biodiesel sample is mixed with 1 mL of hydroxylamine reagent and heated at 60 °C for 20 minutes to ensure complete ester reaction. After being cooled to room temperature, the mixture is acidified with 1 mL of HCl (1 : 1 dilution with water), followed by the addition of 1 mL of ferric chloride reagent. The final volume is adjusted to 10 mL with solvent and thoroughly mixed. The absorbance of the resulting purple complex is measured at 540 nm against the blank, and a calibration curve is plotted (absorbance *vs.* ethyl oleate concentration).

A linear regression of the data provides the calibration equation: *A* = 0.0421*C* + 0.0123, where *A* is the absorbance, and *C* is the ester concentration in mg L^−1^. The high correlation coefficient (*R*^2^ = 0.9911) confirms the reliability of the method.

This method provides a rapid, simple, and nondestructive way to determine ester concentration in biodiesel samples. The EN 14214 standard requires that biodiesel have a minimum ester content of ≥96.5% (w/w) to ensure fuel quality, engine compatibility, and compliance with international regulations. The ester content is calculated using the following equation:1
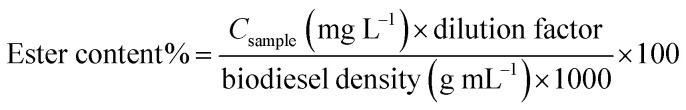


##### 2.2.4.5 Determination of AV and FFA% by colorimetric titration using phenolphthalein

The free fatty acid (FFA) content of the biodiesel samples was determined by a classical colorimetric titration using phenolphthalein as the indicator.^47^ A known mass of the sample (typically 0.50 g) was dissolved in 10 mL of an ethanol/toluene solvent mixture to ensure complete homogenisation. Two to three drops of phenolphthalein indicator were then added, and the mixture was titrated with a standardized alcoholic potassium hydroxide solution (0.10 mol per L KOH), previously calibrated against a primary standard (standard HCl solution) to confirm its exact normality. The titration was conducted under continuous stirring until a faint, persistent pink color appeared for approximately 30 seconds, which was taken as the endpoint. A blank titration, consisting of the solvent and indicator without the sample, was performed in parallel to correct for the background alkalinity of the solvent system. The Acid Value (AV) expressed in mg KOH per g of sample was calculated using:2
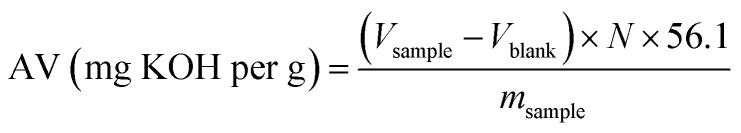
where *V*_sample_ and *V*_blank_ are the volumes of KOH consumed by the sample and of KOH consumed when titrating only the solvent + indicator, without the sample (in mL), *N* is the exact molar concentration of KOH (mol L^−1^), 56.1 is the molecular weight of KOH (in g mol^−1^), and *m*_sample_ is the mass of the sample (g). According to ASTM D664, the acid value in biodiesel should not exceed 0.50 mg KOH per g.

The Free Fatty Acid content (FFA%), expressed as oleic acid, was calculated from the AV using the standard conversion:3
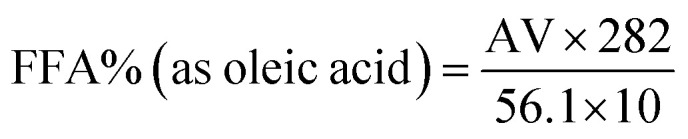
where 282 is the molecular weight of oleic acid (in g mol^−1^).

##### 2.2.4.6 Determination of water content by oven drying

The water content (mg kg^−1^) of the biodiesel samples was also evaluated using a conventional oven-drying (loss-on-drying) method. In this procedure, an accurately weighed portion of biodiesel (2 g) was placed in a pre-dried, pre-weighed glass. The sample was then heated in a ventilated drying oven at 105 ± 2 °C for a fixed period (usually 1 hour) to evaporate free and loosely bound water. After heating, the dish was removed, cooled to room temperature, and reweighed. The water content was calculated from the loss in mass before and after drying and expressed as a percentage of the initial sample mass. The water content is calculated as:4
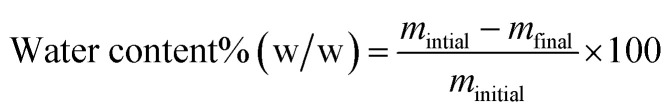


According to ASTM D6304, the water content of biodiesel should not exceed 500 mg kg^−1^.

##### 2.2.4.7 Quantification of retained and free glycerol

UV-vis spectroscopy was used to quantify the concentrations of retained glycerol in biodiesel (Gly_Bio_) and free glycerol (Gly_free_). In contrast, FTIR spectroscopy confirmed the presence of characteristic functional groups, notably hydroxyl (OH) stretching vibrations (3200–3500 cm^−1^), thereby confirming the molecular identities of biodiesel and glycerol. For quantification of Gly_Bio_ and Gly_free_, a colorimetric spectrophotometric protocol involving periodate oxidation and chromogenic derivatization was used.^48^ The samples were treated with sodium periodate (NaIO_4_, 0.1 M) under controlled conditions (37 °C, 15 min) to oxidize glycerol to formaldehyde. The formaldehyde was then reacted with acetylacetone (2% v/v Nash reagent in ammonium acetate buffer, pH 6.0) at 55 °C for 10 min, producing a yellow diacetyldihydrolutidine complex (DDL). Absorbance was measured at *λ*_max_ = 410 nm using a UV-vis spectrophotometer, with deionized water as the blank. A calibration curve (*Y* = 0.042*X* + 0.0267, *R*^2^ = 0.9886) was established using standard glycerol solutions (1–100 mM), enabling quantification by linear equation *C* = (*A* − *b*)/*m*, where *C* is the concentration (mM), *A* is the absorbance, and *b* (intercept) and *m* (slope) are calibration parameters. This method facilitated comparative analysis of concentrations of retained and free glycerol in FeNP-catalyzed reactions. Subsequently, the density (*d* in g cm^−3^) of the free glycerol solutions was calculated using the equation of the calibration curve *d* = 0.998 + 0.002*C*, where *C* is the derived glycerol concentration (mM). This equation, validated for 1–100 mM glycerol, incorporates the baseline density of pure water (0.998 g cm^−3^) and accounts for the linear density increase (0.002 g per cm^3^ per mM glycerol) due to the contribution of glycerol.

The results were used to assess the influence of FeNP characteristics on both the efficiency of the transesterification reaction and the ease of glycerol separation, thus providing insight into the role of nanocatalysts in optimizing biodiesel purification.

##### 2.2.4.8 Calculation of yield and parameters of separation efficiency and their standard deviation

Sustainable production of high-quality biodiesel is dependent on the rigorous evaluation of key performance metrics that collectively ensure efficiency, purity, and regulatory compliance. Among these, the biodiesel yield (*R*_Bio_%), separation efficiency (Sep_Eff_%), variability in separation (SD_Sep_Eff__%) and non-compliant residual components (RNC%) serve as indispensable indicators to assess both the viability of the process and the suitability of the product.

• The biodiesel yield (*R*_Bio_%), expressed as a percentage, is calculated by dividing the mass of purified biodiesel (in grams) by the initial mass of oil feedstock (in grams) and multiplying by 100, as defined in eqn (5):5
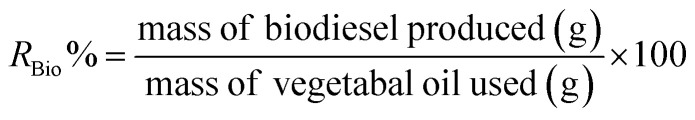


This metric serves as a direct indicator of transesterification efficiency, reflecting the catalyst's ability to convert TGs into fatty acid esters (biodiesel) while minimizing unreacted oil, side reactions (*e.g.*, saponification), or product loss during purification. A higher *R*_Bio_% signifies superior conversion efficiency and catalyst performance, as it approaches the theoretical maximum yield dictated by the stoichiometry of the reaction. Conversely, lower yields highlight inefficiencies such as incomplete conversion, poor phase separation, or emulsification issues. By correlating *R*_Bio_% with catalyst properties, this measurement enables systematic optimization of reaction conditions to maximize feedstock utilization and meet industrial biodiesel quality standards.

• Separation efficiency (Sep_Eff_%) quantifies the effectiveness of glycerol removal from the biodiesel phase after transesterification. It is calculated using the formula:6
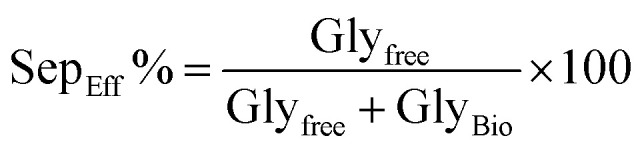
and7
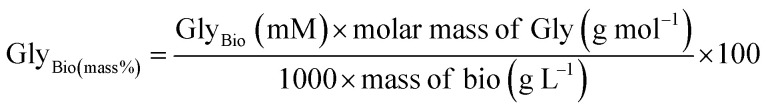
where Gly_Bio_ represents the retained glycerol concentration in biodiesel (determined *via* periodate oxidation/UV-vis) and Gly_free_ is the free glycerol concentration after separation.

High Sep_Eff_% ensures minimal retained glycerol in biodiesel, critical for compliance with fuel standards (*e.g.*, EN 14214 limits glycerol to ≤0.02% w/w). Excess glycerol can impair engine performance, cause injector fouling, or increase emissions. It reflects the efficacy of phase separation methods (*e.g.*, settling and centrifugation) and catalyst performance. Poor Sep_Eff_% indicates emulsification issues or inadequate catalyst-driven destabilization of the glycerol–biodiesel emulsion.

• SD_Sep_Eff__ (Standard Deviation of Separation Efficiency) is a statistical measure that quantifies the variability or dispersion of separation efficiency (Sep_Eff_%) values across multiple experimental trials. It is calculated as:8
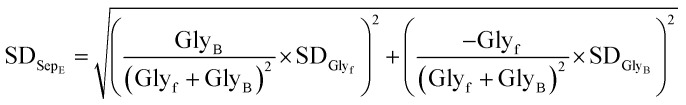
where the calculation of SD_Sep_Eff__ ensures compliance with industrial biodiesel standards (*e.g.*, EN 14214) by validating minimal variability in retained glycerol content, a critical parameter for fuel quality and engine performance. This metric also facilitates catalyst performance evaluation: lower SD_Sep_Eff__ values indicate catalysts capable of achieving consistent phase separation. Conversely, elevated SD_Sep_Eff__ signals potential flaws in experimental design, such as inherent catalyst instability. SD_Sep_Eff__ < 1% is considered excellent, indicating highly reproducible separation with minimal variability. This ensures consistent retained glycerol levels well below the EN 14214 threshold (≤0.02% w/w). Thus, SD_Sep_Eff__ serves as a multifaceted tool for quality assurance, catalyst benchmarking, and process diagnostics in biodiesel synthesis.

• RNC% (Reduction Needed for Compliance) quantifies the percentage reduction required in retained biodiesel bound glycerol (Gly_Bio_) to meet the regulatory limit (*e.g.*, EN 14214: ≤0.02% mass or 1.91 mM or ≤200 µg g^−1^).^49^ It applies only to non-compliant catalysts that exceed the threshold. It is calculated as:9
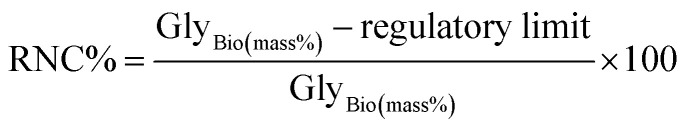


• Over lim% indicates the percentage by which the retained glycerol concentration exceeds the regulatory limit. It applies only to non-compliant catalysts. For compliant catalysts, this metric is irrelevant (marked as none). It is calculated as:10
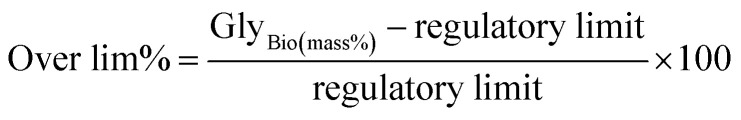


##### 2.2.4.9 Statistical analysis

All data are expressed as mean ± standard deviation (SD) based on three independent replicates. A one-way analysis of variance (ANOVA), followed by Tukey's post hoc test, was performed to evaluate the statistical significance of differences in Gly_Bio_ (mM), *R*_Bio_%, and Sep_Eff_% among the various plant-based FeNP catalysts. A *p*-value < 0.05 was considered statistically significant. All statistical analyses were performed with GraphPad Prism 5 and IBM SPSS Statistics 26 to ensure a rigorous and reliable interpretation of the data.

## 3 Results and discussion

### 3.1 Characterization of plant aqueous extracts

**Fig. 1:**
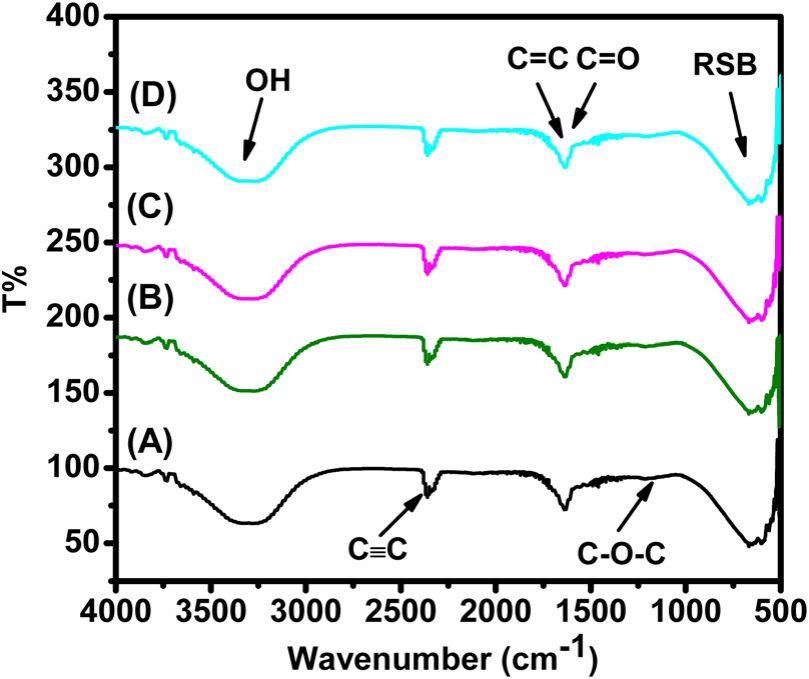
FTIR spectra of plant extracts: (A) *Artemisia herba-alba* (L.), (B) *Juniperus phoenicea* (L.), (C) *Matricaria pubescens* (L.), and (D) *Rosmarinus officinalis* (L.).^44^

Among the FTIR spectra of the four aqueous extracts (Fig. 1), the most notable differences were observed in the vibrational stretching of the –OH groups associated with polyphenols. The peak of *Artemisia herba-alba* extract appeared broadest, followed in order by *Rosmarinus officinalis*, *Matricaria pubescens*, and *Juniperus phoenicea*. These spectral features were consistent with the calculated total polyphenol contents (Table 2), which showed the highest values for *Artemisia herba-alba* (310.84 ± 1.43 mg CAE per g DPE), followed by *Rosmarinus officinalis* (L.) (294.94 ± 0.93 mg CAE per g DPE), *Matricaria Pubescens* (L.) (237.11 ± 1.04 mg CAE per g DPE), and finally *Juniperus phoenicea* (L.) (181.22 ± 1.06 mg CAE per g DPE). These are the most highlighted findings from the phytochemical analysis, which have already been reported in our earlier work^44^ and are briefly summarized here for comparison. Table 2 presents the total contents of polyphenols (TCP), flavonoids (TCF), and condensed tannins (TCCT) in aqueous extracts of four plants: MAT, ROS, JUN, and ARM. These phenolic compounds (flavonoids and condensed tannins) are known to influence the polarity and hydrophilicity of plant extracts. Obtained results reveal that ARM exhibited the highest total polyphenol content (310.84 ± 1.43 mg ACE) and the lowest total flavonoid content (209.50 ± 0.89 mg AGE); however, its condensed tannin content (853.04 ± 0.83 mg CE) was comparable to that of the other extracts. Although ARM's TCF is lower than that of the other extracts, its higher TCP reflects an overall higher polarity of the extract. This suggests that ARM has a composition rich in hydrophilic phenolic structures compared to the three other aqueous extracts, favoring solubility in aqueous media.

In contrast, ROS and MAT extracts, although showing high TCF values (353.75 ± 1.02 and 314.58 ± 1.04 mg AGE, respectively), have comparatively lower TCP-to-TCF ratios, which may reflect a relatively higher proportion of less polar phenolic subclasses that contribute to hydrophobicity. JUN shows intermediate values in all categories, indicating a balanced composition but with less overall polyphenolic richness.

Based on the analysis of TCP, TCF, and TCCT, the degree of hydrophobicity among the aqueous extracts of the plant can be inferred. Extracts with higher proportions of flavonoids relative to polyphenols and lower total polyphenol contents are generally more hydrophobic, as flavonoids often possess more nonpolar characteristics compared to condensed tannins and other water-soluble phenolics.

Among the four plant extracts, ARM shows the highest hydrophilicity due to its high total polyphenol and tannin content, along with the lowest flavonoid content, indicating the least hydrophobic character. Conversely, ROS, with relatively lower polyphenols and higher flavonoids, appears to be the most hydrophobic. Accordingly, the order of hydrophobicity from least to most hydrophobic is: ARM < JUN < MAT < ROS.

### 3.2 Structural, morphological, and optical features of FeNPs

**Fig. 2:**
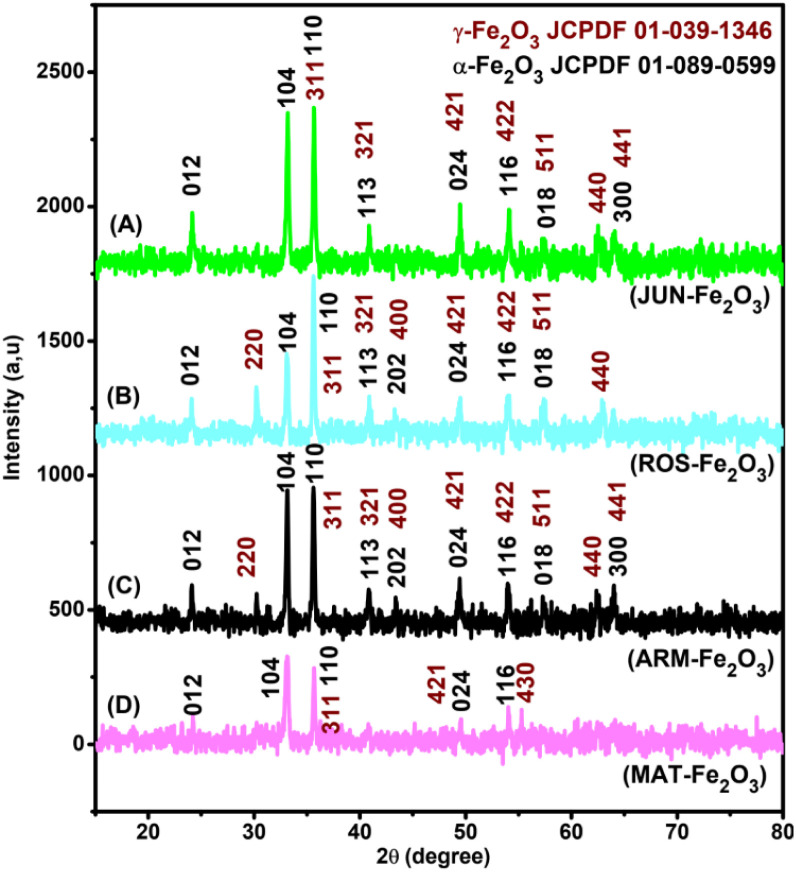
XRD patterns of annealed α/γ-Fe_2_O_3_ samples at 500 °C for 2 h: (A) JUN-α/γ-Fe_2_O_3_, (B) ROS-α/γ-Fe_2_O_3_, (C) ARM-α/γ-Fe_2_O_3_, and (D) MAT-α/γ-Fe_2_O_3_. The diffraction peaks correspond to both the orthorhombic phase of α-Fe_2_O_3_ (JCPDS 01-089-0599) and the cubic phase of γ-Fe_2_O_3_ (JCPDS 00-039-1346).^44^

**Fig. 3:**
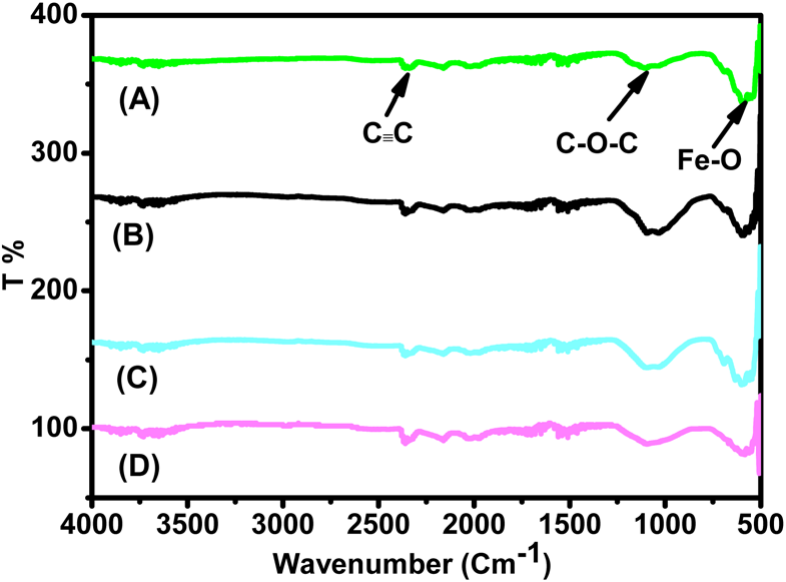
FTIR spectra of FeNPs annealed at 500 °C for 2 h: (A) JUN-α/γ-Fe_2_O_3_, (B) ARM-α/γ-Fe_2_O_3_, (C) ROS-α/γ-Fe_2_O_3_, and (D) MAT-α/γ-Fe_2_O_3_ NPs.^44^

**Fig. 4:**
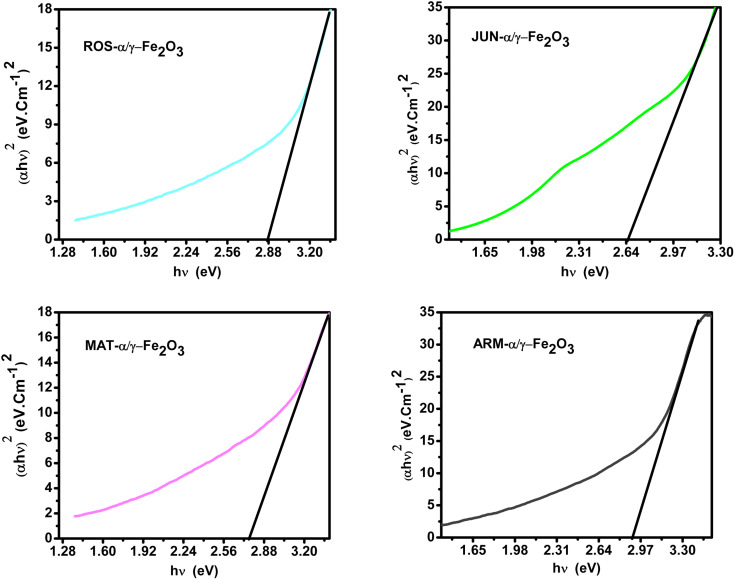
Tauc plots of direct UV-vis transition of annealed plant-based α/γ-Fe_2_O_3_ NPs at 500 °C for 2 h, sonicated in acetone for 15 min.^44^

**Fig. 5:**
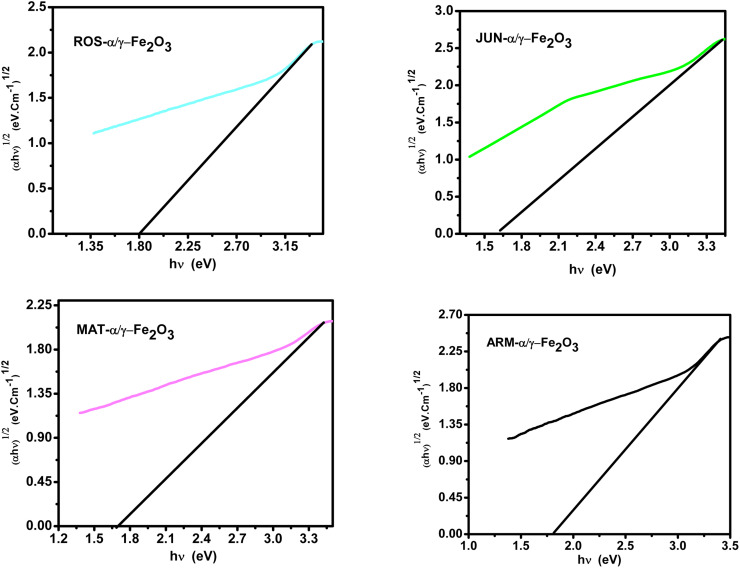
Tauc plots of indirect UV-vis transition of annealed plant-based α/γ-Fe_2_O_3_ NPs at 500 °C for 2 h sonicated in acetone during 15 min.^44^

**Fig. 6:**
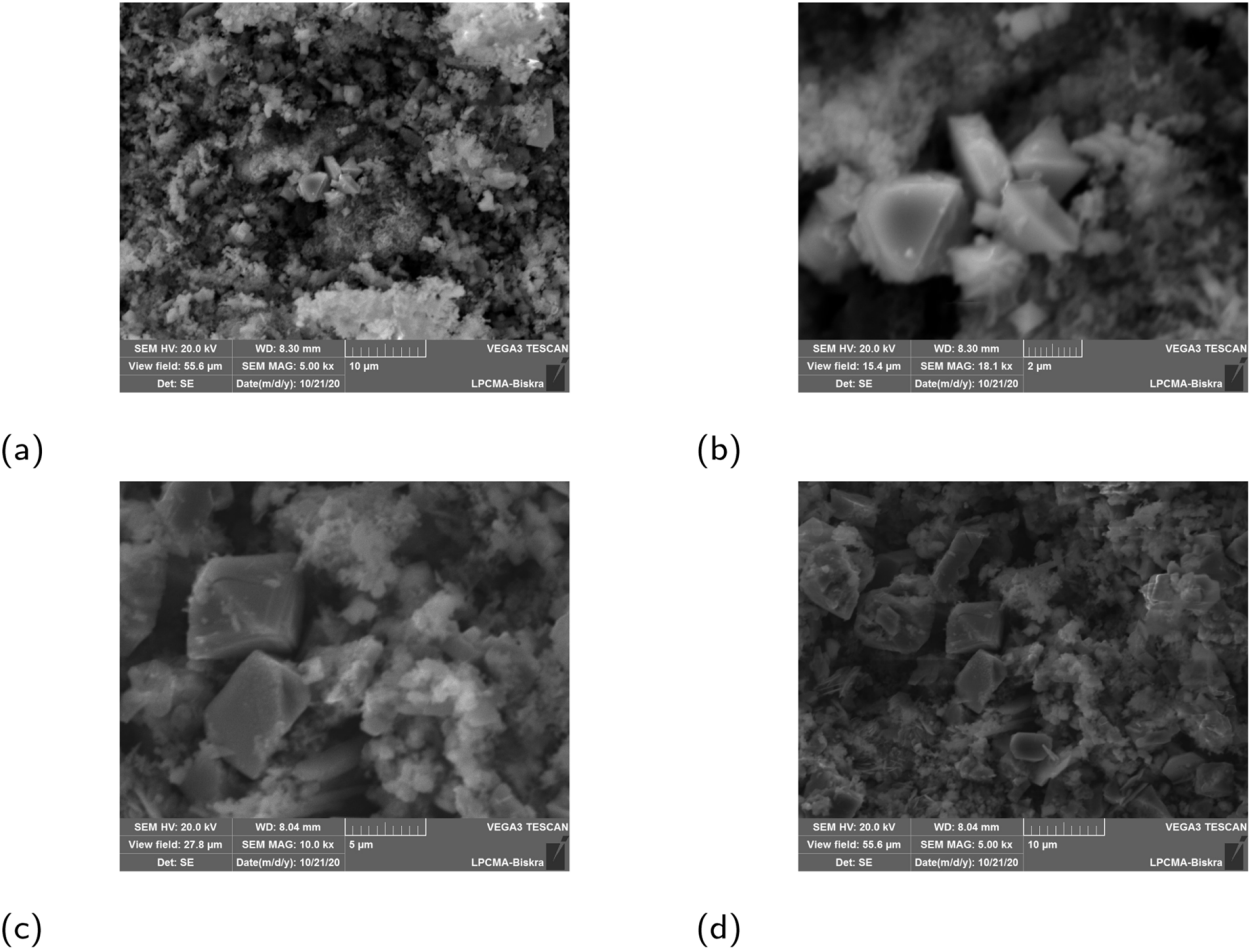
SEM images of green synthesized FeNPs: (a) α/γ-JUN-Fe_2_O_3_, (b) α/γ-MAT-Fe_2_O_3_, (c) α/γ-ROS-Fe_2_O_3_, and (d) α/γ-ARM-Fe_2_O_3_ NPs.^44^

The structural and morphological properties of FeNPs synthesized from different plant extracts were in agreement with our previously published results.^44^ XRD and UV-vis confirmed the presence of mixed α-Fe_2_O_3_ and γ-Fe_2_O_3_ phases with crystallite sizes in the nanometer range. SEM micrographs revealed nearly bi-pyramid particles with moderate agglomeration, while FTIR spectra displayed characteristic Fe–O vibrations and phytochemical-derived functional groups. The results demonstrated that mediating plant extract significantly influenced the physicochemical characteristics of FeNPs, as revealed by XRD, SEM, UV-vis, and FTIR analyses (Fig. 2–6). Extracts with higher antioxidant capacity, such as *Rosmarinus officinalis* and *Artemisia herba-alba*, produced nanoparticles with smaller crystallite size, improved crystallinity, and narrower band gaps, while extracts with lower activity yielded larger, more defect-rich particles. FTIR spectra further confirmed that surface functionalization varied with extract composition, reflecting the distinct phytochemicals present in each plant and their role in stabilizing and capping the FeNPs.

XRD analysis confirmed that all FeNPs synthesized from the four plant extracts contained mixed phases of γ-Fe_2_O_3_ (62–75%) and α-Fe_2_O_3_ (25–38%), with maghemite consistently dominant. Particle sizes were uniform across the samples, with γ-Fe_2_O_3_ crystallites (23.6 nm) smaller than α-Fe_2_O_3_ (29.30 nm), reflecting the larger growth tendency of hematite during annealing. Lattice parameters of γ-Fe_2_O_3_ were close to standard cubic values, while α-Fe_2_O_3_ showed deviations consistent with minor structural defects. Extracts with higher TAC (*Rosmarinus officinalis* and *Artemisia herba-alba*) produced FeNPs richer in the γ-Fe_2_O_3_ phase and with better crystallinity, whereas *Juniperus phoenicea* and *Matricaria pubescens* favored higher α-Fe_2_O_3_ phase content and greater lattice distortions. These findings suggest that TAC plays a more critical role than total polyphenols in determining phase composition and crystallinity. The results were statistically validated by ANOVA and Tukey's test, confirming significant differences among the samples. Detailed crystallographic data and extended discussion have already been published in our earlier work^44^ and are briefly summarized here for context.

In conclusion, the findings indicate that the TAC (presented in Table 2) of plant extracts is a key factor in promoting the crystallinity and stability of the γ-Fe_2_O_3_ phase in FeNPs. Extracts with higher TAC values, such as *Rosmarinus officinalis* and *Artemisia herba-alba*, yielded nanoparticles with improved crystallinity and fewer defects, whereas lower TAC values, as in *Juniperus phoenicea* and *Matricaria pubescens*, were associated with increased defect levels and a higher proportion of the α-Fe_2_O_3_ phase.

The optical band gap analysis confirmed direct gap values between 2.66 eV and 2.91 eV and indirect gap values between 1.61 eV and 1.82 eV for FeNPs synthesized with different plant extracts, in close agreement with literature values.^50–52^ A clear size–band gap correlation was observed, where smaller crystallite sizes corresponded to larger band gaps, reflecting the quantum size effect.^35,53,54^ These are the main outcomes of the structural and optical characterization, while detailed spectra, numerical data, and extended discussion have already been published in our earlier work^44^ and are only briefly summarized here for context.

### 3.3 Green-catalyzed biodiesel production from vegetable oil TGs using phytosynthesized FeNPs

Transesterification is the primary reaction to produce biodiesel from TGs found in vegetable oils. In this process, TGs react with short-chain alcohols such as ethanol to form fatty acid ethyl esters (FAEEs, biodiesel) and glycerol, as detailed in eqn (11)–(15).^55^ When catalyzed by FeNPs (the catalysts compared are ROS-FeNPs, MAT-FeNPs, JUN-FeNPs, and ARM-FeNPs), the reaction benefits from enhanced catalytic efficiency. The FeNPs act as heterogeneous catalysts, offering a green alternative to conventional base or acid catalysts. Their surface properties can be tailored to promote the ethanolysis of TGs, improving yield and selectivity. This approach facilitates cleaner separation of glycerol and enhances the sustainability of biodiesel production.

11 R_1_COOCH_2_,R_2_COOCH,R_3_COOCH_2_ + 3CH_2_CH_3_OH → 3RCOOCH_2_CH_3_ + HO–CH_2_–CHOH–CH_2_OH

• First, FeNPs activate the carbonyl of ester groups:

12 TG + FeNPs → TG* (activated ester)

• First ethanolysis: ethanol attacks activated carbonyl groups:

13 R_1_COO–CH_2_ + CH_2_CH_3_OH → R_1_COOCH_2_CH_3_ + HO–CH_2_

• Second ethanolysis:

14 R_2_COO–CH + CH_2_CH_3_OH → R_2_COOCH_2_CH_3_ + HO–CH

• Third ethanolysis:

15 R_3_COO–CH_2_ + CH_2_CH_3_OH → R_3_COOCH_2_CH_3_ + HO–CH_2_

Post-reaction, the mixture was allowed to settle for 24 h to enable phase separation. Due to differences in density, the free glycerol formed during the transesterification reaction settled at the bottom and was separated from the biodiesel by decantation. Both phases were isolated and prepared for characterization.

The free glycerol and the glycerol that was retained in the biodiesel phase were analyzed using the same oxidative detection approach. In this method, glycerol was first oxidized to formaldehyde using sodium periodate, followed by colorimetric detection with Nash reagent. This two-step process enabled reliable quantification of glycerol in both phases: free glycerol and glycerol retained in the biodiesel phase (unremoved glycerol). By applying the same analytical protocol to both fractions, consistent comparisons could be made to evaluate the efficiency of glycerol removal and separation achieved by different FeNP catalysts.

Biodiesel samples synthesized in the presence of four different FeNPs were rigorously evaluated for key fuel properties, including kinematic viscosity, density, water content, acid value, and FFA%, to assess compliance with international biodiesel standards such as EN 14214, ASTM D93, ASTM D664, and ASTM D6304.

The evaluation of key fuel properties revealed a consistent influence of catalyst hydrophobicity on biodiesel quality. Initial analysis confirmed that all FeNP-catalyzed biodiesel variants exhibited kinematic viscosity (*ν*_bio_) and density (*d*_bio_) within the EN 14214 specification ranges of 1.98–3.12 cSt at 40 °C and 870–890 kg m^−3^ at 15 °C, respectively (Table 5), indicating no adverse alteration of these fundamental characteristics. Extending the characterization to critical safety and purity parameters, a clear gradient emerged. The hydrophobicity of the nanocatalyst directly influenced the kinematic viscosity and density of the final biodiesel by governing the efficiency of glycerol co-product removal. A more hydrophobic surface, as seen in ROS-FeNPs, promotes cleaner phase separation, minimizing the retention of polar glycerol within the biodiesel phase. This results in lower, more optimal values for both kinematic viscosity (1.98 cSt) and density (870 kg m^−3^), as residual glycerol—which is more viscous and dense than biodiesel—is effectively excluded. Conversely, less hydrophobic catalysts (*e.g.*, ARM-FeNPs) lead to higher glycerol retention, resulting in elevated viscosity (3.12 cSt) and density (890 kg m^−3^), approaching the upper limits of the specification range.

**Table 5:** Impact of catalyst type on free glycerol (Gly_free_) separated from the biodiesel phase

Catalyst	*d*_aqu-gly_ (g cm^−3^)	Gly_free_ (mM)	Ester content (%)	*ν*_bio_ (cSt)	*d*_bio_ (kg m^−3^)	*R*_Bio_ (%)	Gly_Bio_ (µg g^−1^)	AV (mg KOH per g)	FFA (%)	Water content (mg kg^−1^)
ROS-FeNPs	1.133 ± 0.08	67.53 ± 0.69	98.25	1.98	870	92.60 ± 1.12	152.70 ± 0.87	0.32 ± 0.02	0.16 ± 0.01	280 ± 20
MAT-FeNPs	1.147 ± 0.05	74.62 ± 0.57	96.52	2.12	876	85.78 ± 1.87	153.00 ± 0.88	0.38 ± 0.03	0.19 ± 0.02	325 ± 25
JUN-FeNPs	1.157 ± 0.01	79.64 ± 3.20	91.50	2.85	883	82.39 ± 1.14	900.00 ± 0.79	0.45 ± 0.03	0.23 ± 0.02	410 ± 30
ARM-FeNPs	1.165 ± 0.03	83.81 ± 4.50	89.09	3.12	890	81.42 ± 2.03	909.40 ± 0.94	0.49 ± 0.04	0.25 ± 0.02	480 ± 35

The flash point (presented in Table 10), a key safety metric, was highest for biodiesel from the most hydrophobic ROS-FeNPs (168 ± 2 °C) and decreased progressively for MAT-FeNPs (162 °C), JUN-FeNPs (155 °C), and ARM-FeNPs (148 °C), though all values substantially exceeded the 101 °C minimum. Conversely, properties linked to impurities and corrosion potential showed an inverse trend. The acid value and FFA content were lowest for ROS-FeNPs (0.32 mg KOH per g; 0.16%) and increased for the less hydrophobic catalysts, with ARM-FeNPs (0.49 mg KOH per g; 0.25%) approaching the specification limit. Most tellingly, water content was minimized to 280 mg kg^−1^ using ROS-FeNPs but increased to 480 mg kg^−1^ with ARM-FeNPs, directly reflecting inferior post-reaction purification. This consistent pattern across physical, safety, and purity parameters confirms that hydrophobic nanocatalysts such as ROS-FeNPs yield biodiesel with superior overall quality and full compliance with international fuel standards, ensuring compatibility with conventional engines.

The characterization of biodiesel phases is essential to evaluate the dual function of phytosynthesized FeNPs in promoting transesterification and facilitating post-reaction purification. In this context, FTIR spectroscopy confirms the formation of biodiesel by identifying characteristic ester carbonyl and aliphatic CH stretching vibrations, while also detecting trace amounts of glycerol retained in biodiesel samples. Meanwhile, UV-vis spectroscopy was performed to quantify the ester content in biodiesel samples. The ester content in biodiesel was determined *via* a hydroxamic acid derivatization method, wherein esters react with hydroxylamine under alkaline conditions to form hydroxamic acids. Subsequently, these derivatives complexed with ferric ions (Fe^3+^) in acidic medium, producing a magenta-colored complex quantified spectrophotometrically at *λ*_max_ = 540 nm. For comparative analysis, UV-vis spectroscopy was concurrently employed to directly quantify ester content by dissolving biodiesel samples in *n*-hexane, a nonpolar solvent that enhances the solubility of FAEEs while suppressing interference from polar contaminants such as free glycerol.

For free and retained glycerol samples, UV-vis provides quantitative evidence of glycerol content, both glycerol retained in biodiesel (Gly_Bio_) and free glycerol (Gly_free_). Here, Gly_free_ serves as a proxy for reaction efficiency (yield), while Gly_Bio_ quantifies purification efficacy (quality). This is achieved by monitoring the absorbance of the yellow chromophore formed from the reaction of formaldehyde, generated by oxidation of glycerol with sodium periodate, with Nash reagent, with the observed *λ*_max_ = 410 nm serving as a marker of the presence of glycerol. To quantify retained and free glycerol, derivatization with diacetyldihydrolutidine (DDL) was used. This approach enables the indirect detection of glycerol through UV-vis spectroscopy by forming a stable chromogenic complex (DDL). The characteristic absorption bands were monitored by using FTIR spectroscopy, including the OH stretching vibration of glycerol (3300 cm^−1^) and the C=O stretching of the DDL–glycerol complex (1740 cm^−1^). Peak intensities were normalized to a reference band (*e.g.*, CH stretching at 2900 cm^−1^) to account for sample concentration variations. This method provided insights into glycerol partitioning between biodiesel and free glycerol phases, correlating with purification efficiency. Together, these techniques offer a comprehensive understanding of how different FeNPs influence both biodiesel quality and glycerol removal, serving as an indicator of phase separation effectiveness.

Table 5 summarizes the performance of four FeNP-based catalysts (ROS, MAT, JUN, and ARM) used in the catalysis of the biodiesel process by comparing the density (*d*, g cm^−3^) of the aqueous phase containing free glycerol, the concentration of free glycerol (Gly_free_, mM), the ester content% in biodiesel samples, and the percentage of biodiesel yield (*R*_Bio_%). Together, these parameters provide information on the efficiency of glycerol removal and the effectiveness of phase separation facilitated by each catalyst.

The free glycerol concentration (Gly_free_) in the ROS-FeNP sample, although the lowest among the four catalysts (67.53 ± 0.69 mM), suggests a different mechanism at play. Rather than indicating poor glycerol extraction, this low concentration points to the ability of the ROS-FeNP catalyst to promote the effective separation of glycerol from biodiesel during the reaction itself. As a result, a substantial portion of glycerol is likely removed early and is not left in the biodiesel phase to separate later during settling. This interpretation is supported by the low glycerol content retained in the ROS-FeNP-biodiesel rich phase (Gly_Bio_ = 1.46 ± 0.21 mM or 152.7 µg g^−1^) and the highest biodiesel yield (*R*_Bio_ = 92.60 ± 1.12%), indicating minimal loss of biodiesel in the aqueous phase. Furthermore, the density (*d*_aqu-gly_) of the aqueous phase follows a pattern similar to that of the free glycerol concentration in the aqueous phase, increasing from 1.133 ± 0.08 g cm^−3^ for ROS-FeNPs to 1.165 ± 0.03 g cm^−3^ for ARM-FeNPs. Since glycerol has a higher density than water, the increase in density reflects a higher glycerol content in the aqueous phase, which validates the previous measurements of Gly_free_. This correlation also reinforces the reliability of using the solution density as a supportive indicator of the presence of glycerol in such aqueous phases.

As evidenced in Table 5, the ester content% (FAEEs) of biodiesel samples exhibited a direct correlation with biodiesel yield, underscoring the critical role of catalytic efficiency in transesterification. ROS-FeNPs, achieving the highest FAEE content (98.25%), aligned with its superior biodiesel yield (92.60 ± 1.12%), reflecting near-complete TG conversion and minimal side-product formation. MAT-FeNPs met the EN 14214 threshold (96.52% FAEEs), correlating with an 85.78 ± 1.87% yield, indicative of compliant purity despite marginally lower catalytic activity. Conversely, JUN-FeNPs (91.50% FAEEs) and ARM-FeNPs (89.10% FAEEs) demonstrated substandard ester content and reduced yields (82.39 ± 1.14% and 81.42 ± 2.03%, respectively), signifying incomplete reactions and inefficient feedstock utilization. These trends confirm that higher FAEEs purity directly corresponds to enhanced catalytic performance and yield, validating ester content as a robust proxy for assessing both reaction completeness and compliance with industrial biodiesel standards.

The findings conclusively demonstrate that the type of FeNP catalysts significantly impacts its performance in biodiesel production, with distinct variations in catalytic activity, phase separation efficiency, and glycerol removal capacity observed between plant-synthesized nanoparticles. This counterintuitive result, where intermediate catalysts outperform ARM-FeNPs despite similar synthesis protocols, underscores the critical role of customized catalyst properties in governing emulsion stability and separation efficacy. Thus, even marginal improvements in the catalyst's properties can significantly enhance phase partitioning, increasing purification, and ensuring compliance with biodiesel quality standards.

In the following section, a comprehensive statistical analysis of Gly_Bio_, *R*_Bio_%, and Sep_Eff_% is performed to evaluate the performance differences in the separation of glycerol between the FeNP catalysts. This analysis is essential to validate each catalyst's sensitivity and reliability, and determine whether the observed differences are statistically significant or simply due to random variation.

#### 3.3.1 Statistic analysis of Gly_Bio_, *R*_Bio_%, and Sep_Eff_%

A one-way analysis of variance (ANOVA) was conducted to determine if the differences in key performance metrics—retained glycerol (Gly_Bio_ mM), separation efficiency (Sep_Eff_%), and biodiesel yield (*R*_Bio_%)—across the four catalysts were statistically significant. The analysis revealed highly significant differences for all three parameters (*p* < 0.0001), confirming that the type of plant extract used in FeNP synthesis had a substantial impact on catalytic performance (Table 6).

**Table 6:** ANOVA results for Gly_Bio_ (mM), Sep_Eff_%, and *R*_Bio_%

Metric	Factor	*d*_f_ (between)	*d*_f_ (within)	*F*-Value	*p*-Value
Gly_Bio_ (mM)	Catalyst	3	8	112.4	<0.0001
Sep_Eff_ (%)	Catalyst	3	8	89.7	<0.0001
*R*_Bio_ (%)	Catalyst	3	8	45.2	<0.0001

One-way ANOVA revealed significant differences in Gly_Bio_, Sep_Eff_%, and *R*_Bio_% across catalysts (*p* < 0.0001). Tukey's HSD post-hoc test delineated performance hierarchies: ROS-FeNPs and MAT-FeNPs outperformed JUN- and ARM-FeNPs in glycerol removal, with Gly_Bio_ values of 1.46 ± 0.21 mM for ROS-FeNPs and 8.69 ± 1.80 mM for ARM-FeNPs (*p* < 0.001), and phase separation efficiency of 98.30 ± 0.01% for ROS-FeNPs and 88.60 ± 0.63% for ARM-FeNPs (*p* < 0.001). However, ROS-FeNPs exhibited the highest *R*_Bio_ = 92.60 ± 1.12%, indicating a trade-off between yield and purification efficiency. MAT-FeNPs balanced these metrics *R*_Bio_ = 85.78 ± 1.87%, while JUN-FeNPs and ARM-FeNPs underperformed due to their hydrophilic tendencies and poor interfacial activity. These results highlight the pivotal role of catalyst hydrophobicity in optimizing transesterification. Hydrophobic surfaces facilitate glycerol exclusion, promote phase separation, and suppress emulsion formation, factors that collectively improve fuel quality. For industrial standards such as EN 14214, ROS-FeNPs and MAT-FeNPs emerge as the most suitable catalysts. This analysis supports the strategic design of catalysts with enhanced hydrophobicity to balance yield, purity, and scalability in biodiesel production (Tables 7–9).

**Table 7:** Tukey's test for retained glycerol Gly_Bio_ (mM)

Comparison	Mean difference (mM)	95% CI	Adjusted *p*-value	Significance
ROS *vs.* ARM	+7.23	[6.12, 8.34]	<0.001	Highly significant
MAT *vs.* ARM	+7.00	[5.89, 8.11]	<0.001	Highly significant
ROS *vs.* JUN	+5.21	[4.10, 6.32]	<0.001	Highly significant
JUN *vs.* MAT	+4.98	[3.87, 6.09]	<0.001	Highly significant
JUN *vs.* ARM	−2.02	[−3.13, −0.91]	<0.001	Highly significant
MAT *vs.* ROS	−0.23	[−1.34, 0.88]	0.12	Not significant

**Table 8:** Tukey's test for separation efficiency Sep_Eff_%

Comparison	Mean difference (%)	95% CI	Adjusted *p*-value	Significance
ROS *vs.* ARM	−9.70	[−10.2, −9.2]	<0.001	Highly significant
MAT *vs.* ARM	−9.30	[−9.8, −8.8]	<0.001	Highly significant
ROS *vs.* JUN	−6.50	[−7.0, −6.0]	<0.001	Highly significant
JUN *vs.* MAT	−6.10	[−6.6, −5.6]	<0.001	Highly significant
MAT *vs.* ROS	−0.40	[−0.90, +0.10]	0.09	Not significant
JUN *vs.* ARM	+3.20	[2.7, 3.7]	0.02	Not significant

**Table 9:** Tukey's test for biodiesel yield *R*_Bio_%

Comparison	Mean difference (%)	95% CI	Adjusted *p*-value	Significance
ROS *vs.* ARM	+11.18	[8.5, 13.9]	<0.001	Highly significant
MAT *vs.* ARM	+4.36	[1.6, 7.1]	0.03	Not significant
ROS *vs.* JUN	+10.21	[7.5, 12.9]	<0.001	Highly significant
JUN *vs.* MAT	+3.39	[0.7, 6.1]	0.04	Not significant
MAT *vs.* ROS	+6.82	[4.1, 9.5]	<0.001	Highly significant
JUN *vs.* ARM	+0.97	[−1.7, 3.7]	0.12	Not significant

These trends correspond to the total content of flavonoid (TCF) and total condensed tannin content (TCCT) of the plant extracts used in the synthesis of the FeNPs. Extracts from *Rosmarinus officinalis* and *Matricaria pubescens*, which contain higher levels of TCF and TCCT, yielded FeNPs with catalytic properties that hindered free glycerol from being separated from the organic phase. In contrast, extracts from *Juniperus phoenicea* and *Artemisia herba-alba*, characterized by lower TCF and TCCT, produced FeNPs that facilitated more effective glycerol separation.

### 3.4 Impact of green nanocatalyst type on biodiesel production and purification

**Fig. 7:**
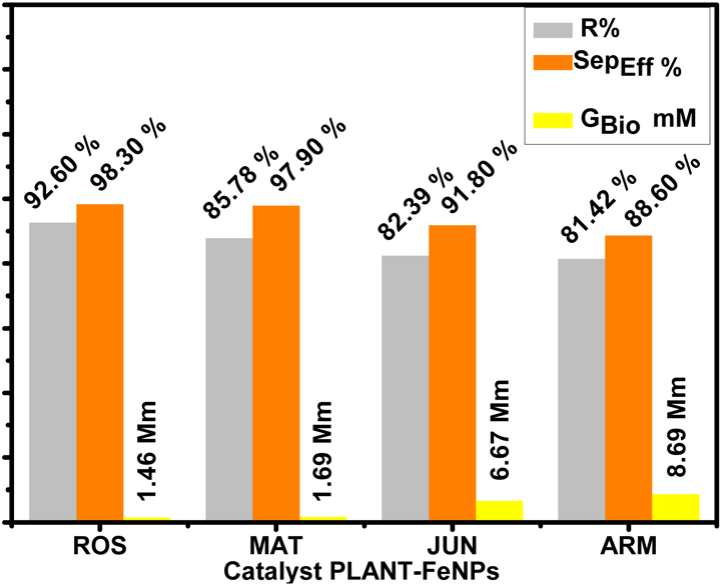
Impact of FeNP type on biodiesel yield and purification.

The comparative evaluation of FeNP-based catalyst performance, presented in Table 10 and Fig. 7, reveals significant differences in the efficiency of glycerol separation and the purity of biodiesel. ROS-FeNPs and MAT-FeNPs exhibit notably low retained glycerol concentrations in the biodiesel phase, with Gly_Bio_ measured to be 1.46 ± 0.21 mM and 1.69 ± 0.14 mM, respectively. These values correspond to mass fractions of just 0.0153% and 0.0177%, respectively, which are well within the acceptable threshold for fuel-grade biodiesel according to the EN 14214 limit (≤1.91 mM). In contrast, JUN-FeNPs and ARM-FeNPs demonstrate significantly higher retained glycerol contents, Gly_Bio_ = 6.67 ± 0.69 mM and 8.69 ± 0.32 mM, respectively, indicating incomplete separation of glycerol during the reaction.

**Table 10:** Influence of FeNP green nanocatalyst type on biodiesel yield and glycerol retained in biodiesel (Gly_Bio_) *vs.* EN 14214 limit: ≤0.02% mass or ≤1.91 mM

Catalyst	Gly_Bio_ (mM)	Gly_Bio mass_ (% mass)	Compl. status	Over limit	RNC (%)	Sep_Eff_ (%)	SD_Sep_Eff__ (%)	Flash point (°C)	Fire point (°C)
ROS-FeNPs	1.46 ± 0.21	0.0153	Yes	None	None	98.30 ± 0.01	0.02	168 ± 2	182 ± 2
MAT-FeNPs	1.69 ± 0.14	0.0177	Yes	None	None	97.90 ± 0.03	0.03	162 ± 3	176 ± 3
JUN-FeNPs	6.67 ± 1.50	0.0695	None	247.5	71.30% red	91.80 ± 0.54	1.20	155 ± 2	168 ± 2
ARM-FeNPs	8.69 ± 1.80	0.0909	None	353.5	77.50% red	88.60 ± 0.63	1.50	148 ± 3	160 ± 3

In line with this, only ROS-FeNPs and MAT-FeNPs meet the international glycerol purity standards, achieving a “Yes” status for compliance, with no exceedance of the allowable limits. JUN-FeNPs and ARM-FeNPs, however, exceed the limit by 247.50% and 353.50%, respectively. This deviation is further quantified by the Reduction Need Coefficient (RNC)%, which shows that these two nanocatalysts require more than 70% glycerol reduction to meet the standards (71.30% for JUN-FeNPs and 77.50% for ARM-FeNPs), underscoring the inefficiency of their separation mechanisms. Furthermore, separation efficiency (Sep_Eff_%) further confirms this trend. ROS-FeNPs (98.30 ± 0.01%) and MAT-FeNPs (97.90 ± 0.03%) achieve a near-complete removal of glycerol, validating their strong post-reaction purification potential. In contrast, JUN-FeNPs (91.80 ± 0.54%) and ARM-FeNPs (88.60 ± 0.63%) exhibit considerably lower efficiencies, aligned with their high retained glycerol levels and non-compliant status. Furthermore, ROS-FeNPs and MAT-FeNPs exhibit exceptional consistency in glycerol removal, with minimal variability (SD_Sep_Eff__ = 0.02–0.03%) and high separation efficiencies (Sep_Eff_ = 97.9–98.3%). These catalysts ensure compliance with EN 14214 standards (retained glycerol 0.0153% and 0.0177%, respectively), indicating their stable process performance. In contrast, JUN-FeNPs (SD_Sep_Eff__ = 1.2% and Sep_Eff_ = 91.80%) and ARM-FeNPs (SD_Sep_Eff__ = 1.5% and Sep_Eff_ = 88.6%) show significant variability, leading to non-compliance (retained glycerol: 0.0695% and 0.0909% mass, respectively) and excessive loss of biodiesel. Their instability comes from fluctuations in the process, resulting in inefficient emulsion breakdown. The trend in biodiesel yield also reflects the influence of glycerol separation. ROS-FeNPs yielded the highest biodiesel production at 92.60 ± 1.12%, followed by MAT-FeNPs at 85.78 ± 1.87%. Conversely, ARM-FeNPs and JUN-FeNPs yielded 81.42 ± 2.03% and 82.39 ± 1.14%, respectively, the lowest among the tested samples. This correlation suggests that ineffective removal of glycerol likely impairs the equilibrium of the transesterification reaction, thus limiting the overall conversion and yield.

These findings demonstrate the superior catalytic and purifying performance of ROS- and MAT-derived FeNPs, with ROS-FeNPs outperforming others in biodiesel yield, glycerol removal, and compliance with fuel quality standards. Thus, the differences in catalytic behavior are not purely chemical but also involve physicochemical interactions during phase separation, governed by the structural and surface properties of FeNP catalysts, which are influenced by their green synthesis route.

These findings suggest that ROS-FeNPs facilitate early and clean phase separation, contributing to efficient transesterification and product recovery. In contrast, ARM-FeNPs, with reduced catalytic performance (81.42 ± 2.03% yield), retained significantly higher glycerol (8.69 ± 0.32 mM or 909.4 µg g^−1^), reflecting incomplete reaction and poor phase partitioning. Intermediate catalysts such as JUN-FeNPs (*R*_Bio_ = 82.39 ± 1.14) and MAT-FeNPs (85.78 ± 1.87%) exhibit a balance between moderate glycerol recovery and acceptable biodiesel retention. Their performance suggests partial mitigation of emulsification, likely due to specific surface properties that enable limited destabilization of the biodiesel–glycerol emulsion. In contrast, ARM-FeNPs' notably lower catalytic efficiency (*R*_Bio_ = 81.42 ± 2.03%) and poor phase separation (Gly_Bio_ = 8.69 ± 0.32 mM) highlight insufficient surface reactivity to disrupt the emulsion, leaving phases incompletely resolved. This difference may be attributed to surface hydrophobicity, a key factor in controlling emulsion stability and phase separation during transesterification. ROS-FeNPs appear to possess more hydrophobic surface properties, which facilitate the destabilization of the biodiesel–glycerol emulsion and promote more efficient phase separation. Their surface likely limits glycerol adsorption and encourages its exclusion from the nonpolar biodiesel phase. In contrast, ARM-FeNPs exhibit lower catalytic efficiency, suggesting insufficient surface hydrophobicity and poor emulsion-breaking capability, resulting in incomplete separation of product phases.

UV-vis and FTIR spectroscopy provide essential tools for evaluating biodiesel quality, particularly in monitoring retained and free glycerol content after transesterification. Free glycerol, if not efficiently separated, compromises biodiesel purity and engine performance, while retained glycerol bound to the catalyst surface reflects interactions at the bio–catalyst interface. By applying spectroscopic techniques to quantify both forms, it becomes possible to directly assess how the hydrophobicity of the FeNP nanocatalysts influence biodiesel production and purification. More hydrophobic catalysts are expected to interact less with glycerol, leading to lower retained levels in the biodiesel phase, whereas more hydrophilic catalysts may promote higher retention. Thus, in the next section, spectroscopic analysis of glycerol will provide a reliable means of linking catalyst properties, biodiesel yield, and post-reaction purification efficiency.

#### 3.4.1 Spectroscopic characterization of post-biodiesel samples: evaluating yield and purity of biodiesel *via* UV-vis and FTIR analyses

**Fig. 8:**
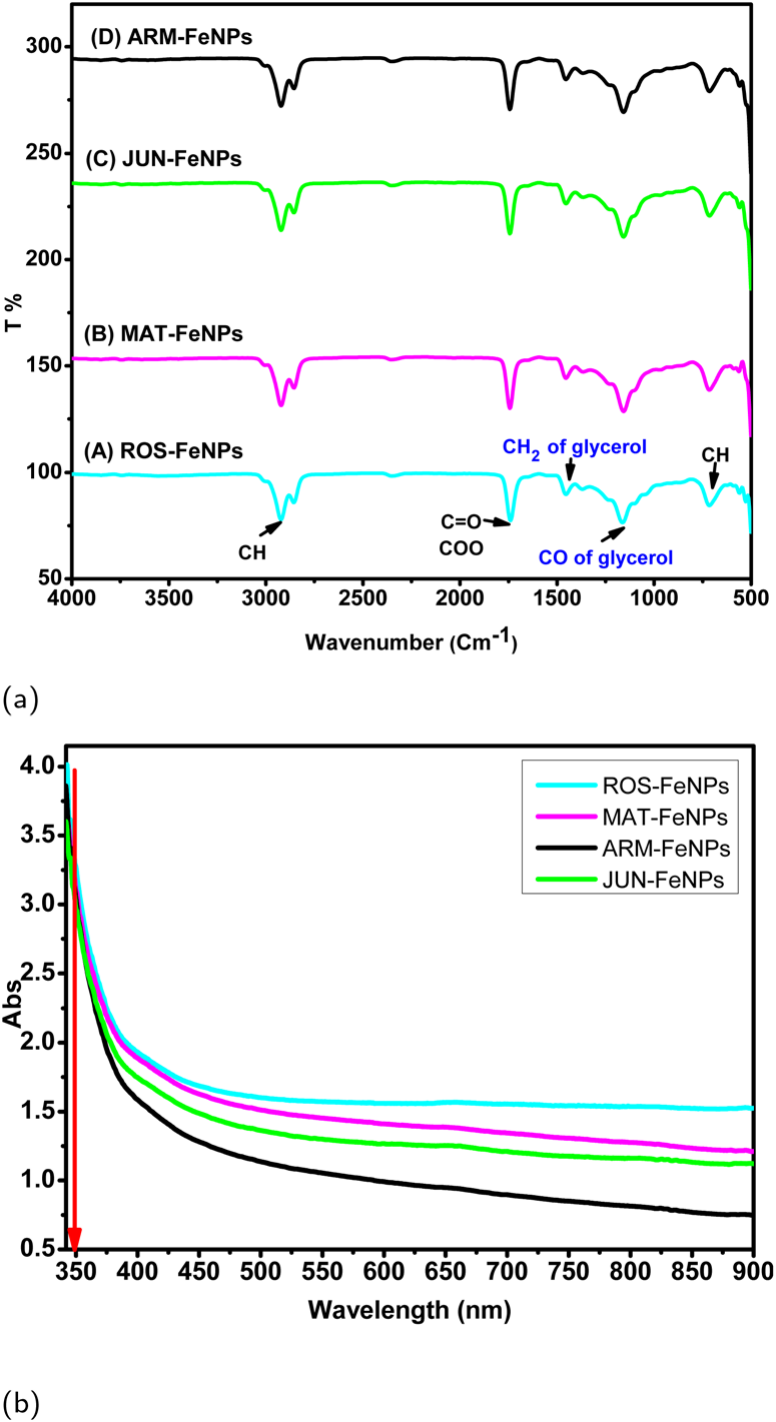
(a) IR spectra of biodiesel produced with various phytosynthesized catalysts: (A) ROS-FeNPs, (B) MAT-FeNPs, (C) JUN-FeNPs, and (D) ARM-FeNPs. (b) UV-vis spectra of biodiesel produced with these catalysts.

Fig. 8a shows the FTIR spectra of biodiesel derived from the ethanolic transesterification of TGs, catalyzed by four different phytosynthesized FeNPs: (A) ROS-FeNPs, (B) MAT-FeNPs, (C) JUN-FeNPs, and (D) ARM-FeNPs. The spectra highlight key functional groups associated with FAEEs, the main components of biodiesel, as well as signals indicative of retained glycerol within the biodiesel phase presented by DDL absorption peaks. Notable differences in peak intensity and area across the samples suggest that the FeNPs exert distinct influences on both the catalytic transesterification efficiency and the subsequent phase separation of glycerol. Fourier-transform infrared (FTIR) spectroscopy of the biodiesel phases confirmed successful transesterification, as evidenced by the characteristic ester carbonyl (C=O) stretching band at approximately 1736 cm^−1^ and aliphatic C–H stretching vibrations near 2900 cm^−1^ (Fig. 8a). A clear gradient in the intensity of the carbonyl band was observed, with ROS-FeNPs and MAT-FeNPs exhibiting the most intense signals, followed by JUN-FeNPs and ARM-FeNPs. This visual trend in peak intensity aligns with the quantified ester content values (98.25% to 89.09%, Table 5), where a more intense C=O band corresponds to a higher concentration of fatty acid ethyl esters (FAEEs). A smaller band near 700 cm^−1^, associated with CH bending in unsaturated FAEEs, reflects compositional variability in biodiesel products. Similarly, Fig. 8b depicts the UV-vis spectra of biodiesel samples. All spectra exhibit a distinct absorbance maximum at *λ*_max_ = 350 nm, characteristic of conjugated systems in FAEEs, confirming successful biodiesel formation.^56,57^

Importantly, the relative intensities of the ester carbonyl peaks at 1736 cm^−1^ vary across the FeNP samples. ROS-FeNPs show the most intense and broadest peak, followed by MAT-FeNPs, whereas JUN-FeNPs and ARM-FeNPs exhibit narrower and weaker peaks. Since the peak area is proportional to ester content, this observation aligns well with the quantitative data in Table 10. The analysis of ester content in the four biodiesel samples revealed significant quality variations. The ester content was quantified using the calibration curve (*A* = 0.0421*C* + 0.0123), where *C* represents ester concentration (mg mL^−1^). Samples of biodiesel produced in the presence of ROS-FeNPs and MAT-FeNPs demonstrated exceptionally high ester content of 98.25% and 96.52%, respectively, surpassing the stringent threshold of the EN 14214 standard (minimum requirement: 96.50%), indicating optimal transesterification efficiency. In contrast, samples JUN-FeNPs and ARM-FeNPs exhibited subpar ester content of 91.50% and 89.09%, respectively, suggesting incomplete conversion of TGs during biodiesel production. This trend correlates directly with catalytic performance: ROS-FeNPs and MAT-FeNPs exhibited superior catalytic activity, driving near-complete conversion and yielding the highest yield: *R*_Bio_ = 92.60 ± 1.12% and 85.78 ± 1.87%, respectively, and the highest purity: Gly_Bio_ = 1.46 ± 0.21 mM and 1.69 ± 0.14 mM, respectively. The results highlight the critical role of catalyst efficiency in optimizing transesterification. Enhanced catalytic systems, such as ROS-FeNPs and MAT-FeNPs, not only improve conversion yields but also ensure compliance with industrial benchmarks, underscoring their potential for scalable biodiesel production.

Further evidence of retained glycerol with biodiesel appears in the form of DDL absorption bands at 1450 cm^−1^ (C–O–H bending) and 1140 cm^−1^ (C–O stretching). These bands are significantly more intense in biodiesel produced using JUN-FeNPs and ARM-FeNPs, suggesting inefficient glycerol removal during the reaction. This correlates with their higher Gly_Bio_ (6.67 ± 1.50 mM and 8.69 ± 1.80 mM), lower Sep_Eff_ (91.80 ± 0.54% and 88.60 ± 0.63%), and higher SD_Sep_Eff__ (1.2% and 1.5%), as reported in Table 10. In contrast, these bands are much weaker in the ROS-FeNP and MAT-FeNP spectra, indicating more effective post-reaction purification.

In summary, the FTIR data reinforce the findings from quantitative glycerol analysis and biodiesel yield. ROS-FeNP and MAT-FeNP catalysts not only promote higher transesterification efficiency, as reflected in stronger ester signals, but also enable superior glycerol separation, evidenced by weaker glycerol-associated bands. On the other hand, ARM-FeNP and JUN-FeNP catalysts exhibit lower catalytic performance and purification capability, consistent with the spectral features and performance metrics previously discussed.

#### 3.4.2 FTIR and UV-vis spectroscopic characterization of separated free glycerol post-biodiesel

**Fig. 9:**
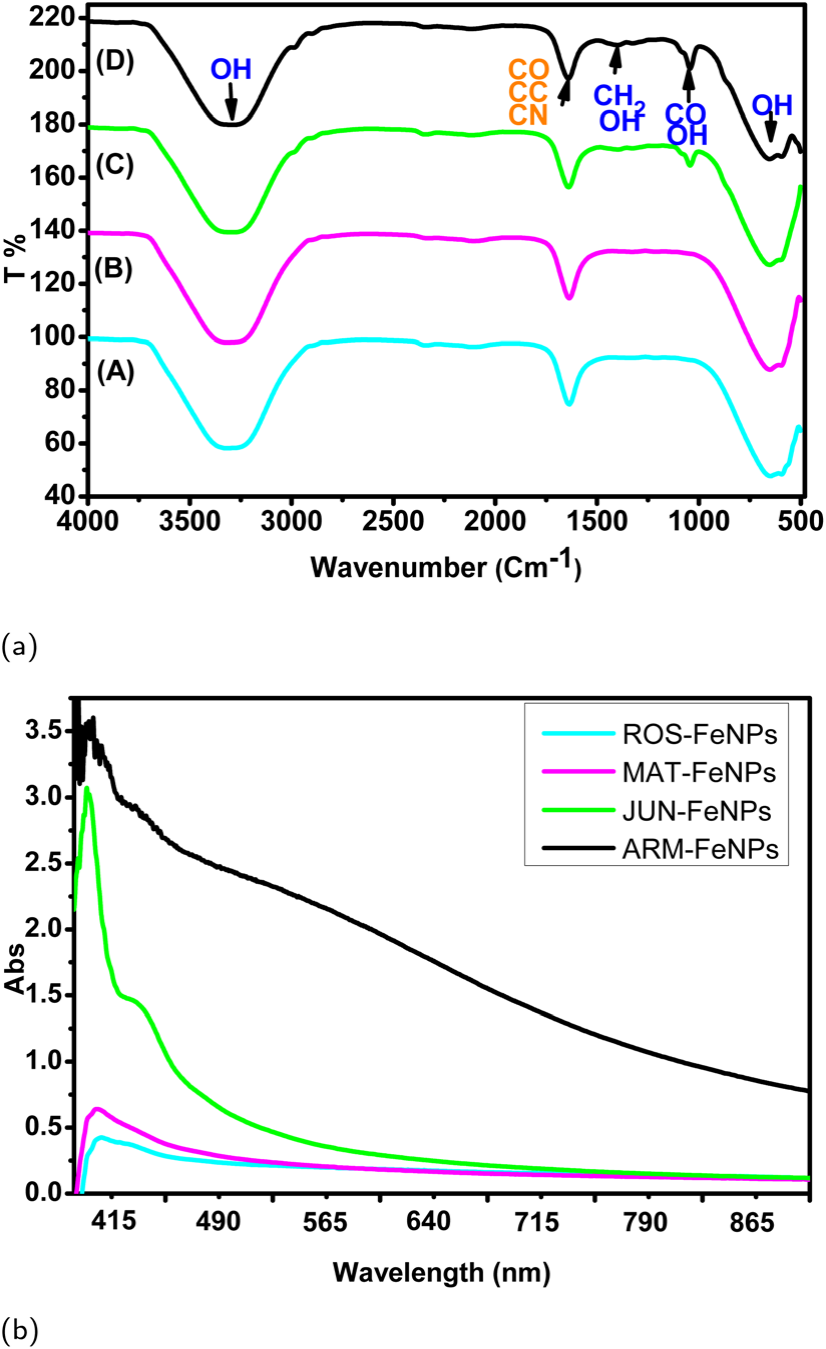
(a) IR spectra of free glycerol separated post-reaction of biodiesel production in the presence of various catalysts: (A) ROS-FeNPs, (B) MAT-FeNPs, (C) JUN-FeNPs, and (D) ARM-FeNPs. (b) UV spectra of glycerol produced with various catalysts: ROS-FeNPs, MAT-FeNPs, JUN-FeNPs, and ARM-FeNPs.

Fig. 9a presents the FTIR spectra of free glycerol collected after biodiesel purification. This analysis evaluates glycerol separation efficiency and confirms the formation of DDL. Key absorbance bands associated with glycerol and DDL were used to compare the concentration of free glycerol in the four aqueous phases.

The broad OH stretching band observed at 3300 cm^−1^ in the FTIR spectra originates from free glycerol in the aqueous phases. This assignment is corroborated by the gradient in band intensity, which aligns with measured Gly_free_ concentrations: strongest in ARM-FeNPs (83.81 ± 0.21 mM) and weakest in ROS-FeNPs (67.53 ± 0.32 mM). Furthermore, the oxidative pathway is further validated by distinct DDL-specific bands in ROS-FeNP and MAT-FeNP spectra: 1700 cm^−1^ for C=O stretching of the acetyl groups in DDL and 1620 cm^−1^ for conjugate C=N and C=C vibrations of the lutidine ring. The broader DDL peaks observed in ROS-FeNPs and MAT-FeNPs, compared to those of ARM-FeNPs and JUN-FeNPs, stem from differences in the amount of free glycerol and its subsequent oxidation dynamics. ROS-FeNPs and MAT-FeNPs exhibit lower concentrations of free glycerol in the aqueous phase (67.53 ± 0.69 mM and 74.62 ± 0.57 mM, respectively), allowing complete oxidation of free glycerol to DDL during analysis. This full conversion generates more pronounced and broader DDL peaks. In contrast, ARM-FeNPs and JUN-FeNPs retain significantly higher free glycerol in the aqueous phase (83.81 ± 4.50 mM and 79.64 ± 3.20 mM, respectively), overpowering the oxidation capacity. The excess glycerol is only partially converted to DDL, resulting in narrower peaks because of the coexistence of unreacted glycerol and limited DDL formation. This disparity highlights how superior phase separation in ROS/MAT-FeNPs minimizes aqueous-phase glycerol, ensuring complete oxidation. In contrast, the inefficient separation of ARM/JUN-FeNPs leaves excess glycerol unresolved, directly impacting DDL peak profiles.

Additionally, the FTIR spectra provide critical insight into the concentration of free glycerol and its oxidative conversion to formaldehyde, which reacts with the Nash reagent to form DDL. In ARM-FeNPs and JUN-FeNPs, prominent bands at 1400 cm^−1^ (C–H_2_ bending and in plane OH bending of glycerol) and 1030 cm^−1^ (CO stretching/out-of-plane OH bending) confirm more free glycerol in the aqueous phase of ARM-FeNPs and JUN-FeNPs. These bands are absent in ROS-FeNPs and MAT-FeNPs, indicating near-complete oxidation of free glycerol to formaldehyde during the reaction. Furthermore, the decreasing 635 cm^−1^ band (out of plane OH bending) in the sequence ARM > JUN > MAT > ROS reflects a gradient in free glycerol concentration, aligned with the phase separation efficiency: Sep_Eff_ = 88.60 ± 0.63% for ARM-FeNPs *vs.* 98.30 ± 0.01% for ROS-FeNPs.

The catalytic efficiency in the separation of the glycerol–biodiesel phase is intrinsically related to the hydrophobicity of the catalyst.^39,58,59^ This relationship suggests that ROS-FeNPs and MAT-FeNPs possess enhanced surface hydrophobicity, which promotes preferential partitioning of glycerol during transesterification. In contrast, ARM-FeNPs and JUN-FeNPs exhibit lower hydrophobicity, stabilizing glycerol within the organic (biodiesel) phase. This retention reduces the accessibility of glycerol for downstream derivatization reactions, thereby reducing the overall efficiency of the process. The divergent behavior underscores the critical role of tailored catalyst hydrophobicity in optimizing phase separation and ensuring complete glycerol removal, essential for high purity biodiesel production.

Fig. 9b displays the UV-vis spectra of the four aqueous phases containing free glycerol. A prominent absorbance peak at *λ*_max_ = 410 nm, characteristic of DDL, confirms the presence of derivatives derived from free glycerol in the aqueous phase. The absorbance intensity correlates directly with the free aqueous glycerol concentration: ARM-FeNPs, with the highest Gly_free_ = 83.81 ± 4.5 mM, exhibit the strongest absorption, while ROS-FeNPs, with the lowest Gly_free_ = 67.53 ± 0.32 mM, show the weakest absorption. This trend reflects the divergent efficiencies of the catalysts in separating glycerol during transesterification. Most hydrophobic catalysts (ROS-FeNPs and MAT-FeNPs) minimize glycerol retention in the biodiesel phase, leading to lower Gly_free_ and reduced DDL formation. In contrast, less hydrophobic catalysts (ARM-FeNPs and JUN-FeNPs) stabilize glycerol in the biodiesel phase, yielding stronger Gly_free_ and DDL absorbance. The 410 nm peak thus serves as a quantitative proxy for glycerol partitioning, validating UV-vis as a robust tool for assessing both derivatization efficiency and phase separation performance in biodiesel systems.

### 3.5 Impact of mediating plant extract on the transesterification process and purification

The catalytic performance of four phytosynthesized FeNPs during ethanol mediated transesterification of TGs exhibited marked variations in biodiesel yield (81.42 ± 2.03% to 92.60 ± 1.12%), ester content (89.09–98.25%), and glycerol separation efficiency (98.30–88.60%), underscoring the critical role of catalyst design in reaction outcomes. These results align with prior reports,^3,10,60^ emphasizing that altering the catalyst governs the transesterification reaction and phase separation dynamics.The physicochemical properties of catalysts directly influence biodiesel yield and purity, as evidenced by variations in aqueous glycerol (Gly_free_) and retained biodiesel bound glycerol Gly_Bio_. ROS-FeNP and MAT-FeNP catalysts enhance interfacial contact between ethanol and TGs, driving lower Gly_free_ (67.53 ± 0.69 mM and 74.62 ± 0.57 mM, respectively), revealing efficient transesterification and glycerol partitioning into the aqueous phase. This correlates with elevated biodiesel yields (92.60 ± 1.12%) and minimal Gly_Bio_ (1.46 mM and 1.67 mM), ensuring compliance with EN 14214 standards (<0.02% mass). Conversely, ARM-FeNP and JUN-FeNP catalysts exhibit lower Gly_free_ (83.81 mM and 79.64 mM) and elevated Gly_Bio_ (8.69 mM and 6.67 mM), reflecting incomplete reactions and poor phase separation, which degrade fuel quality. The hydrophobicity of catalysts is pivotal in biodiesel synthesis,^39,58,59^ as it governs interfacial interactions between polar alcohols such as ethanol and nonpolar triglycerides, minimizes water-induced side reactions, and enhances phase separation of glycerol. However, a key question arises: how does plant extract, employed in the green synthesis of FeNPs, influence catalyst hydrophobicity?

**Fig. 10:**
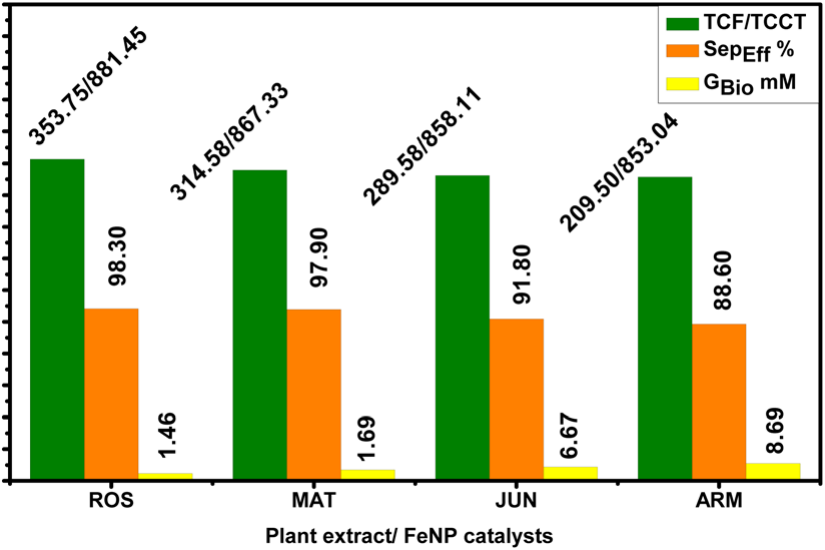
Impact of mediating plant extract hydrophobicity on plant-based FeNP performance in biodiesel yield and purification.

The hydrophobicity of phytofabricated FeNPs is intrinsically tied to the biochemical profile of the plant extract used in their synthesis. Plant extracts rich in hydrophobic phytochemicals, such as flavonoids and condensed tannins, act as dual reducing and capping agents during FeNP formation. These hydrophobic moieties adsorb onto nanoparticle surfaces, creating a nonpolar capping layer that enhances the hydrophobicity of FeNPs. For instance, Table 11 and Fig. 10 show that ROS-FeNPs, synthesized using a plant extract with a high TCF/TCCT content, exhibited superior hydrophobicity, enabling efficient separation of glycerol during transesterification and adsorption at the triglyceride–ethanol interface. This hydrophobic capping minimizes water intrusion (reducing hydrolysis side reactions) and promotes preferential interaction with nonpolar TGs, accelerating transesterification kinetics. Consequently, ROS-FeNPs achieved the highest biodiesel yield (92.60 ± 1.12%), highest ester content (98.25%), and lowest retained glycerol (Gly_Bio_ = 1.46 ± 0.21 mM) compared to ARM-FeNPs derived from less hydrophobic extracts, which achieved the lowest biodiesel yield (81.42 ± 2.03%), lowest ester content (89.09%), and highest retained glycerol (Gly_Bio_ = 8.69 ± 1.80 mM). These findings underscore that plants with abundant hydrophobes serve as green templates for tailoring the surface properties of FeNPs, optimizing their catalytic activity and interfacial dynamics for biodiesel production and purification. Thus, the biochemical composition of mediating plant extracts directly dictates the hydrophobicity of FeNPs, underscoring the need for a strategic selection of phytochemical-rich species to engineer high-performance catalysts for sustainable biodiesel systems.

**Table 11:** Correlation between extract hydrophobicity properties and glycerol separation efficiency in biodiesel purification using FeNP catalysts

Extract/FeNPs	TCF (mg AGE)	TCCT (mg CE)	Gly_free_ (mM)	Gly_Bio_ (mM)	Sep_Eff_ (%)	*R*_Bio_ (%)	Ester content (%)
ROS	353.75 ± 1.46	881.45 ± 1.02	67.53 ± 0.69	1.46 ± 0.21	98.30 ± 0.01	92.60 ± 1.12	98.25
MAT	314.58 ± 0.80	867.33 ± 1.04	74.62 ± 0.57	1.69 ± 0.14	97.90 ± 0.03	85.78 ± 1.87	96.52
JUN	289.58 ± 1.12	858.11 ± 0.93	79.64 ± 1.02	6.67 ± 0.69	91.80 ± 0.54	82.39 ± 1.14	91.50
ARM	209.50 ± 0.86	853.04 ± 0.89	83.81 ± 1.04	8.69 ± 0.32	88.60 ± 0.63	81.42 ± 2.03	89.09

The most significant finding of this work is the direct role of catalyst hydrophobicity in governing the efficiency of post-reaction purification. We propose that the hydrophobic capping layer, derived from flavonoids and tannins in the plant extract, creates a non-polar surface on the FeNPs. This surface exhibits low affinity for the highly polar glycerol molecules. Consequently, during transesterification, catalysts such as ROS-FeNPs and MAT-FeNPs act as phase-transfer agents that repel glycerol, facilitating its expulsion from the biodiesel phase and promoting the formation of a distinct, separable glycerol layer. This results in high separation efficiency (Sep_Eff_ > 97.9%) and minimal retained glycerol (Gly_Bio_ ≤ 1.91 mM). In contrast, the more hydrophilic surfaces of ARM-FeNPs and JUN-FeNPs stabilize glycerol at the oil–catalyst interface, leading to emulsion formation, incomplete phase separation, and higher glycerol contamination in the final biodiesel product. This mechanistic insight directly links green synthesis parameters to a critical industrial processing advantage.

The spectroscopic data provide compelling evidence for the proposed mechanism. The FTIR spectra of biodiesel (Fig. 8a) not only confirm transesterification but also serve as a qualitative indicator of purity. The attenuated glycerol-associated OH bands and weaker DDL-related signals in the ROS-FeNP and MAT-FeNP biodiesel spectra visually corroborate the low Gly_Bio_ values measured quantitatively. Conversely, the UV-vis analysis of the free glycerol phases (Fig. 9b) reveals an inverse relationship: the higher absorbance of the DDL complex at 410 nm for ARM-FeNPs and JUN-FeNPs indicates a greater concentration of glycerol in the aqueous phase, which is a direct consequence of its poor initial separation from biodiesel. Together, these techniques paint a consistent picture: hydrophobic catalysts produce a cleaner biodiesel phase and a more concentrated, separable glycerol by-product, streamlining the entire production process.

### 3.6 Comparative analysis of nanocatalysts in biodiesel production

Table 12 presents a comparative analysis of various nanocatalysts used in the transesterification of different vegetable oils for biodiesel production, focusing on the molar ratio of alcohol to oil, catalyst loading, and resulting yield. Among all catalysts, MgO/Mg–Fe_2_O_4_ achieved the highest biodiesel yield of 95.43% using canola oil, but required a high alcohol-to-oil ratio of 12 : 1 and a catalyst loading of 6.45 wt%.

**Table 12:** Comparison of biodiesel production from vegetable oils in the presence of different catalysts

Nano-catalyst	Feedstock oil	Alcohol/oil ratio	Amount of catalyst (wt%)	Yield (%)	REF
MgO/Mg–Fe_2_O_4_	Canola	12 : 1	6.45	95.43	61
ROS-FeNPs	Sunflower	3 : 1	0.20	92.60	This study
TiO_2_/ZnO	Of palm	6 : 1	2.00	90.00	62
Zn–Mg–Al hydrotalcites	Neem	10 : 1	7.5	90.50	63
MAT-FeNPs	Sunflower	3 : 1	0.20	87.78	This study
Fe/Sn oxide	Soybean	—	—	84.00	64
CuO/Mg	Sunflower	6 : 1	0.25	82.83	65
JUN-FeNPs	Sunflower	3 : 1	0.20	82.39	This study
ARM-FeNPs	Sunflower	3 : 1	0.20	81.42	This study
Ag_2_O	*Prunus bokhariensis* seed	12 : 1	3.5	80.00	66

In contrast, the ROS-FeNP catalyst developed in this study demonstrated a similarly high yield of 92.60% from sunflower oil, with significantly milder conditions: only a 3 : 1 methanol-to-oil ratio and a catalyst loading of 0.20 wt%. Other green synthesized FeNP catalysts from this study, including MAT-FeNPs, JUN-FeNPs, and ARM-FeNPs, also achieved competitive yields of 87.78%, 82.39%, and 81.42%, respectively, under the same low-load and low-ratio conditions. These findings emphasize the high catalytic efficiency and sustainability of green-synthesized FeNPs, particularly ROS-FeNPs, which outperformed several conventional catalysts despite operating under much milder conditions. For example, Zn–Mg–Al hydrotalcites required a 10 : 1 alcohol/oil ratio and 7.5 wt% catalyst to achieve a 90.50% yield, while TiO_2_/ZnO yielded 90% using palm oil with a 6 : 1 ratio and a 2.00 wt% catalyst. Other catalysts such as Fe/Sn oxide, CuO/Mg, and Ag_2_O gave lower yields, further supporting the effectiveness of plant-mediated FeNPs.

In general, the results validate the strong influence of the mediating plant extract on catalytic performance and highlight the potential of green-synthesized FeNPs as efficient, low-cost, and environmentally friendly alternatives for biodiesel production.

### 3.7 Reusability study

**Fig. 11:**
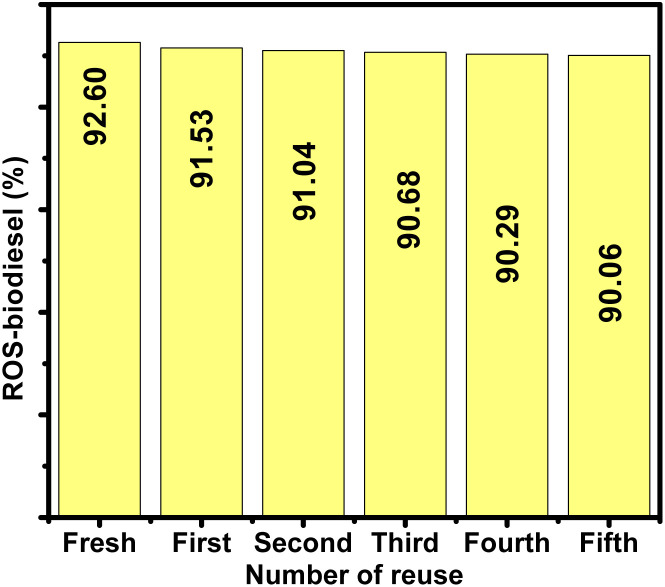
Reusability study of the ROS-FeNP nanocatalyst for five cycles.

The reusability of the ROS-FeNP catalyst was evaluated over five consecutive transesterification cycles to assess its practical stability and economic viability. The catalyst demonstrated exceptional consistency, with the biodiesel yield remaining above 90% throughout all cycles, as presented in Fig. 11. The fresh cycle achieved a yield of 92.60%. After each cycle, the catalyst was recovered *via* centrifugation and regenerated by washing with ethanol and hexane to effectively eliminate adsorbed glycerol, fatty acids, and other reaction impurities from its active sites. This simple regeneration protocol successfully restored catalytic activity, resulting in sustained high yields of 91.53%, 91.04%, 90.68%, 90.29%, and 90.06% for the first, second, third, fourth, and fifth cycles, respectively. The minimal loss in activity (<2% over five runs) confirms the robust nature of the ROS-FeNP catalyst and its strong potential for repeated use in cost-effective, sustainable biodiesel production.

### 3.8 Cost assessment of the ROS-FeNP nanocatalyst and biodiesel

This economic analysis evaluates the commercial viability of the biodiesel synthesis process using the green synthesized ROS-FeNP nanocatalyst. The final production cost is determined by accounting for all expenses, including the cost of sunflower oil, ethanol, and each stage of the catalyst and biodiesel production. The successful commercialization of this transesterification technique hinges on both the high catalytic performance and the favorable cost profile of the ROS-FeNP catalyst. Key factors influencing the catalyst's cost-effectiveness—such as the use of low-cost *Rosmarinus officinalis* biomass, the simple preparation method, and, most importantly, its reusability—are thoroughly assessed. This cost calculation validates the sustainability of the process by considering the catalyst's entire life cycle. The total cost of the ROS-FeNP nanocatalyst, TC^ROS-FeNPs^ (USD per kg), is calculated using a comprehensive formula adapted from the literature,^67,68^ with the detailed breakdown provided in the subsequent equations.

The total cost of synthesizing 1 kg of the ROS-FeNP nanocatalyst (denoted as TC^ROS-FeNPs^) is calculated using the formula:

16 TC^ROS-FeNPs^ = NC^ROS-FeNPs^ + *C*^AE^_ROS-FeNPs_

where NC^ROS-FeNPs^ and *C*^AE^_ROS-FeNPs_ represent net cost of the ROS-FeNP nanocatalyst (USD per kg) and the cost of additional expenses of the ROS-FeNP nanocatalyst (USD per kg), respectively.

The net cost is further broken down as:

17 NC^ROS-FeNPs^ = *C*^ROS^ + UC^ROS^ + UC^ROS-D^ + UC^P^ + UC^EL^ + UC^NPS^ + UC^CH^

where *C*^ROS^: cost of raw material, UC^ROS^: unit cost of *Rosmarinus officinalis* leaves (USD per kg), UC^ROS-D^: unit cost of ROS drying (USD per kg), UC^P^: pyrolysis cost (electricity), UC^EL^: extraction cost (electricity), UC^NPS^: nanoparticle synthesis cost (electricity), and UC^CH^: chemical cost (Fe salts, *etc.*)

18 UC^ROS^ = UC^W^ × *Q*^W^_ROS_

19 UC^ROS-D^ = UC^E^ × *Q*^E^_D_

20 UC^P^ = UC^E^ × *Q*^E^_P_

21 UC^NPS^ = UC^E^ × *Q*^E^_EL_ + UC^E^ × *Q*^E^_NPS_ + UC^E^ × *Q*^E^_R_

22 UC^CH^ = *C*^CH^ × *Q*^CH^

where *Q*^W^_ROS_: water consumed during the washing process (per kg of plant), *Q*^E^_D_: electricity consumed in drying (kWh per kg of plant), *Q*^E^_P_: electricity consumed during pyrolysis (kWh per kg of plant), *Q*^E^_EL_: electricity consumed during extraction of liquid (kWh per kg of plant), *Q*^E^_NPS_: electricity consumed during nanoparticle formation (kWh per kg of plant), and *Q*^CH^: quantity of chemicals (per kg of biodiesel).

The total cost of synthesizing 1 kg of biodiesel (denoted as TC^biodiesel^) is calculated using the formula:

23 TC^ROS-biodiesel^ = NC^ROS-biodiesel^ + *C*^AE^_ROS-biodiesel_

where NC^ROS-biodiesel^ and *C*^AE^_ROS-biodiesel_ represent the net cost of biodiesel production and additional expenses, respectively.

The net cost is further broken down as:

24 NC^ROS-biodiesel^ = *C*^sunflower^_BD_ + UC^ROS-FeNPs^_BD_ + UC^ethanol^_BD_ + UC^trans^_BD_

where *C*^sunflower^_BD_: cost of sunflower oil, UC^ROS-FeNPs^_BD_: catalyst cost per kg biodiesel, UC^ethanol^_BD_: ethanol cost, and UC^trans^_BD_: transesterification electricity cost.

25 UC^ROS-FeNPs^_BD_ = TC^ROS-FeNPs^ × *Q*^ROS-FeNPs^_BD_

26 UC^ethanol^_BD_ = *Q*^ethanol^_BD_ × *C*^ethanol^

27 UC^trans^_BD_ = UC^E^ × *Q*^E^_trans_

where *Q*^ROS-FeNPs^_BD_: quantity of the ROS-FeNP nanocatalyst (kg), *Q*^ethanol^_BD_: quantity of ethanol needed (per kg of biodiesel), *C*^ethanol^: cost of ethanol (USD per kg), and *Q*^E^_trans_: quantity of electricity consumed during transesterification (USD per kg).

The cost of ROS-biodiesel, TC^ROS-biodiesl^ (USD per kg), can be obtained from the developed equation (eqn (16)); this equation has been explained in eqn (18)–(22). Based on our calculations for the scaled-up production of biodiesel using the recyclable ROS-FeNP catalyst, the base production cost is $1.01 per kg. The final cost of the ROS-FeNP catalyst is $6.538 per kg. Notably, the catalyst is reprocessed five times, significantly reducing its effective contribution to the total cost. Furthermore, the catalyst's hydrophobic properties facilitate easier separation of glycerol, reducing purification expenses and providing a cost saving of approximately $0.09 per kg of biodiesel. After accounting for this gain and including a 10% surcharge for large-scale logistics, the final expenditure for 1 kg of biodiesel is calculated to be $1.12. This analysis confirms the commercial viability of using green-synthesized nanocatalysts for sustainable biodiesel production.

## 4 Conclusion

This study elucidates the pivotal role of plant-mediated hydrophobicity in optimizing biodiesel production through phytofabricated FeNPs. By synthesizing FeNPs using aqueous extracts of *Rosmarinus officinalis* (ROS), *Matricaria pubescens* (MAT), *Juniperus phoenicea* (JUN), and *Artemisia herba-alba* (ARM), the work demonstrates that the hydrophobicity of plant-derived phytochemicals, specifically flavonoids and condensed tannins, directly enhances catalytic performance. ROS-FeNPs, enriched with the highest total flavonoid (358.34 ± 1.46 mg AGE per g) and tannin (871.45 ± 0.89 mg CE per g) contents, achieved superior biodiesel yield (92.60 ± 1.12%) and purification efficiency (Sep_Eff_ = 98.30 ± 0.01%), while minimizing retained glycerol in biodiesel (Gly_Bio_ = 1.46 ± 0.21 mM). In contrast, ARM-FeNPs, with the lowest hydrophobicity (209.50 ± 0.89 mg AGE per g; 853.04.50 ± 0.83 mg CE per g), yielded reduced conversion (81.42 ± 2.03%) and poorer phase separation (Sep_Eff_ = 88.60 ± 0.63%), while retaining higher glycerol in biodiesel (Gly_Bio_ = 8.69 ± 1.80 mM), underscoring the inverse relationship between hydrophobicity and emulsification.

The rigorous analytical framework combining FTIR, UV-vis spectroscopy, and aqueous-phase density measurements validated these trends. Attenuated OH glycerol bands and enhanced diacetyldihydrolutidine (DDL) signals in ROS-FeNPs and MAT-FeNPs confirmed efficient glycerol derivatization and phase partitioning. Statistical analyses (ANOVA, *p* < 0.0001; Tukey's HSD) further solidified the superiority of ROS-FeNPs and MAT-FeNPs, grouping them as high-performance catalysts distinct from JUN-FeNPs and ARM-FeNPs. ROS-FeNPs and MAT-FeNPs complied with the EN 14214 standard (Gly_Bio_ ≤ 1.91 mM or ≤200 µg g^−1^), highlighting their industrial viability. Comprehensive spectroscopic and statistical analyses validated these trends, showing consistent attenuation of glycerol-associated OH bands and strong separation of catalyst performance groups under ANOVA and Tukey's HSD tests. The findings highlight hydrophobicity as a critical design parameter in green nanocatalyst engineering, offering a mechanistic basis for improving both reaction efficiency and post-reaction purification.

This study demonstrates that the hydrophobicity of plant extracts is a decisive factor in crafting high-performance FeNP catalysts for biodiesel production. The hydrophobic phytochemicals, primarily flavonoids and condensed tannins, create a non-polar capping layer that confers a dual advantage: it enhances catalytic activity for transesterification, leading to high yields and ester content, and critically, it promotes spontaneous and efficient glycerol phase separation, reducing post-reaction purification burdens. This direct link between green synthesis chemistry and downstream process efficiency represents a significant advancement. By strategically selecting hydrophobic plant species such as *Rosmarinus officinalis*, it is possible to design nanocatalysts that integrate production and purification, offering a more sustainable and economically feasible path to EN 14214-compliant biodiesel.

## Author contributions

Kaouthar Ahmouda: writing-original draft, resources, investigation, visualization, methodology, and revision.

## Conflicts of interest

There are no conflicts to declare.

## Data Availability

The datasets supporting the findings of this study, including the raw experimental measurements (biodiesel yields, physicochemical parameters, UV-vis and FTIR results, and analytical data), are available from the corresponding author upon reasonable request.
